# Pan-cancer analysis of PIEZO1: a promising biomarker for diagnosis, prognosis, and targeted therapies

**DOI:** 10.3389/fimmu.2025.1625734

**Published:** 2025-09-04

**Authors:** Qian Wang, Yan Yu, Xiao Liang, Dan Wan, Ke Du, Pengpeng Zhu

**Affiliations:** ^1^ National‐Local Joint Engineering Research Center of Biodiagnosis & Biotherapy, The Second Affiliated Hospital, Xi’an Jiaotong University, Xi’an, China; ^2^ The Precision Medical Institute, The Second Affiliated Hospital, Xi’an Jiaotong University, Xi’an, China; ^3^ Department of Pathology, The Affiliated Taizhou People’s Hospital of Nanjing Medical University, Taizhou, China

**Keywords:** PIEZO1, pan-cancer, diagnostic biomarker, prognostic biomarker, bioinformatic analysis

## Abstract

**Introduction:**

Piezo-type mechanosensitive channel component 1 (PIEZO1), a mechanically gated cation channel involved in calcium signaling, has been recognized as a potential oncogene in some cancers. However, its comprehensive pan-cancer role remains unexplored.

**Methods:**

This study used The Cancer Genome Atlas (TCGA) data to analyze PIEZO1 expression profiles. Diagnostic value was evaluated using Receiver Operating Characteristic (ROC) curve analysis, with primary tumor samples as cases and adjacent normal tissues as controls. Prognostic value was determined through Cox regression and Kaplan-Meier survival analyses. Clinical correlations were detected using non-parametric tests and logistic regression. Genomic alterations were identified via the cBioPortal database. Functional pathways were analyzed using the R language. The association between PIEZO1 and tumor microenvironment scores (Stromal, Immune, ESTIMATE) or immune checkpoint markers (CD274, CTLA4, LAG3, PDCD1, PDCD1LG2) were analyzed using the R language. Quantitative Real-Time PCR (qRT-PCR) and Western Blotting (WB) were performed to quantify PIEZO1 expression in clinical specimens. Colony formation and wound healing assays assessed PIEZO1’s *in vitro* effects on cancer cells, while xenograft models evaluated its *in vivo* impact on tumor growth.

**Results:**

Our analysis revealed that PIEZO1 has diagnostic and prognostic value across different cancer types. Elevated PIEZO1 expression was associated with advanced tumor grade and stage. Genomic alterations in PIEZO1 were found in 4% of pan-cancer patients. Functional enrichment analyses revealed that PIEZO1-coexpressed genes were significantly enriched in ECM-receptor signaling, cell migration, and ion homeostasis. PIEZO1 positively correlated with tumor microenvironment scores and immune checkpoints. Experimental validation confirmed PIEZO1 overexpression in LIHC and its pro-tumorigenic role *in vitro*/*in vivo*.

**Conclusion:**

Our findings indicate that PIEZO1 is a promising marker for the diagnosis, prognosis, and development of targeted pan-cancer therapies.

## Introduction

Primary liver cancer, ranking sixth in global cancer diagnoses and third in cancer-related deaths, is primarily caused by Liver Hepatocellular Carcinoma (LIHC), which accounts for over 90% of cases (1). The increasing incidence of LIHC is attributed to a complex interplay of factors, including chronic hepatitis virus infections, excessive alcohol consumption, and metabolic syndrome (2–4). Despite medical advancements that have improved the diagnosis and treatment of LIHC, the prognosis remains unfavorable (5). Therefore, it is crucial to gain a deeper understanding of LIHC mechanisms and develop novel therapeutic approaches to improve prognosis.

Piezo-type mechanosensitive channel component 1 (PIEZO1), the first identified mechanically gated cation channel, is present in mammals, mediating the flow of Ca2+ into cells (6). It plays a crucial role in diverse physiological processes, including lymphatic vessel development, axon growth, vascular development, immune modulation, and blood pressure maintenance. In addition to these, PIEZO1 markedly affects cancer development, progression, and invasion, with increasing studies suggesting that its abnormal expression correlates with invasiveness and metastasis of cancer cells.

For example, Mechanical stretch triggers PIEZO1 activation, which in turn promotes the metastasis of cholangiocarcinoma cells through the Hippo/YAP signaling pathway (7). YAP signaling stimulates PIEZO1 expression, modulating oral squamous cell carcinoma cell proliferation (8). PIEZO1 activation contributes to matrix stiffness-induced angiogenesis and metastasis in LIHC (9). PIEZO1 iteratively interacts with glioma tissue mechanics, facilitating malignant progression (10). PIEZO1 is overexpressed in pancreatic cancer tissues compared to normal pancreas, and its high expression correlates with a poor prognosis for patients. Silencing PIEZO1 suppresses the proliferation, migration, and invasiveness of pancreatic cancer cells (11). PIEZO1 fosters prostate cancer progression by activating the Akt/mTOR pathway and accelerating the cell cycle (12). PIEZO1 is upregulated in ovarian cancer tissues, promoting tumor growth and metastasis (13). PIEZO1 sustains the tumorigenic and stemness of CCSCs through the Ca2+/NFAT1 signaling pathway (14). PIEZO1 plays an essential role in regulating the migration and invasion of breast cancer cells by modulating cell mechanobiological properties (15). PIEZO1 enhances the invasion and migration of cervical cancer cells by releasing extracellular ATP (16). Despite these insights, a comprehensive pan-cancer analysis of PIEZO1’s role in various cancers is still needed.

For PIEZO1, we systematically analyzed public databases to detect its expression patterns, diagnostic and prognostic significance, mutational characteristics, biological roles, co-expression patterns, immune interactions, and responses to immunotherapy and drugs. Our analysis revealed that PIEZO1 promotes oncogenic processes across various cancers. This was further supported by molecular biology experiments in LIHC, which verified the contribution of PIEZO1 to tumorigenesis and progression in both *in vitro* and *in vivo* studies. In conclusion, our pan-cancer analysis suggests that PIEZO1 is a potential biomarker associated with tumor growth and may act as a candidate for cancer therapy.

## Materials and methods

### Data preparation and processing

We downloaded the RNA sequencing data and clinical information of PIEZO1 from the TCGA database (https://www.cancer.gov/). To ensure data homogeneity, only primary solid tumors (sample type code: 01) and adjacent normal tissues (sample type code: 11) were retained, while recurrent, metastatic, and other non-primary samples were excluded. For patients with multiple primary tumor samples, the mean expression value was calculated to represent a single data point. The “ggplot2” package in R language facilitated our visualization of the differences. We detected the mRNA expression levels of PIEZO1 across different cancer cell lines based on the HPA database (https://www.proteinatlas.org/) (17).

### Tumor purity assessment and adjustment

To quantify the impact of non-tumor cellular components within the tumor microenvironment, we performed an analysis using the ESTIMATE package in R software (18). This method calculates a Stromal Score, an Immune Score, and a combined ESTIMATE Score for each tumor sample based on its gene expression profile. In subsequent statistical modeling, the ESTIMATE Score was included as a key covariate to account for the potential confounding effects of tumor purity on the analytical results.

### The diagnostic and survival analysis

To evaluate the diagnostic value of PIEZO1 in pan-cancer, we utilized the "pROC" package for analysis and "ggplot2" for visualization in R language. To evaluate the predictive efficiency of PIEZO1 in 1-, 3-, and 5-year OS rates of LIHC patients, we utilized the "timeROC" package for analysis and "ggplot2" for visualization in R language. To evaluate the independent prognostic value of PIEZO1, we constructed multivariate Cox proportional hazards regression models using the R survival package. To account for potential confounding effects from different factors, we implemented two distinct multivariate Cox regression analysis strategies:

Tumor purity-adjusted model: Employing the formula Surv(time, event) ~ PIEZO1_expression + ESTIMATEScore, this model incorporated the ESTIMATE Score as a covariate to effectively control for potential confounding effects of immune and stromal cell infiltration within the tumor microenvironment on prognosis assessment.

Age-adjusted model: Utilizing the formula Surv(time, event) ~ PIEZO1_expression + age, this model included age as a key clinical covariate to eliminate its confounding effect on survival outcomes.

This dual adjustment strategy systematically mitigated the influence of major confounding factors, thereby enabling a more precise assessment of the independent contribution of PIEZO1 expression levels to patient prognosis. All analyses were performed for three critical survival endpoints: Overall Survival (OS), Disease-Specific Survival (DSS), and Disease-Free Interval (DFI). Only cancer types where the sample size and event count met the minimum statistical requirements were included in the final analysis.

### Relationship between PIEZO1 and clinical features

We assessed the relationship between PIEZO1 and a range of clinical and pathological markers (tumor status, histologic grade, ER statuses, PR statuses, histological type, IDH status, pathologic stage, pathologic N stage, pathologic T stage, residual tumor, and metastasis) in cancers using Wilcoxon or Kruskal-Wallis test. In LIHC, we used logistic regression analysis to analyze the association of PIEZO1 and clinical features, including pathologic T stage, pathologic N stage, pathologic M stage, pathologic stage, tumor status, gender, age, BMI, residual tumor, histologic grade, AFP(ng/ml), Albumin(g/dl), prothrombin time, Child-Pugh grade, fibrosis Ishak score, vascular invasion, and adjacent hepatic tissue inflammation.

### Mutation characteristics of PIEZO1

We analyzed the genetic alteration landscape, frequencies, and sites of PIEZO1 in pan-cancer by using the cBioPortal database (https://www.cbioportal.org) (19). We processed and analyzed the MAF file using the R package maftools (20). Tumor Mutational Burden (TMB) was calculated as the total number of non-synonymous somatic mutations per megabase (Mb). Samples were divided into high- and low-expression groups based on the median expression level of PIEZO1. Differentially mutated genes between these two groups were also identified and visualized using this package.

### Relationship between PIEZO1 and m6A-related genes

We downloaded RNA-seq data and corresponding clinical information from the TCGA database. The m6A-associated genes were from the study by Juan Xu et al. (21) We then employed the “ggplot2” and “pheatmap” packages in R language to explore the correlation between PIEZO1 and these m6A-related genes.

### Relationship between PIEZO1 and immune-related factors

Using Spearman’s correlation analysis, we analyzed the correlations between PIEZO1 expression and TMB/MSI scores from TCGA. Using Spearman’s correlation, we also assessed PIEZO1 associations with TIME components (immune cells, stimulators, inhibitors, chemokines, and receptors) and immune checkpoints. Using the “ESTIMATE” package in R language, we examined PIEZO1 associations with stromal cells, as well as immune cells.

### Differentially expressed genes, Gene Ontology, and Gene Set Enrichment Analysis analysis

Differential expression analysis between high and low PIEZO1 expression groups in TCGA-LIHC patients was performed using the limma-voom pipeline (R package limma, v3.50.0) with TMM normalization. Differentially expressed genes (DEGs) were defined as those with |log_2_(fold change)| > 1 and adjusted p-value (FDR) < 0.05. The DEGs were then visualized using “ggplot2” in R. The differentially expressed genes underwent GO and GSEA analysis via the “clusterProfiler” package in R language. Utilizing the c5.all.v7.5.1.symbols.gmt [Gene ontology] gene set, we explored the biological processes and cellular components of PIEZO1. We considered FDR < 0.25 and adjusted p-value < 0.05 as thresholds for statistical significance.

### Investigating the co-expression partners of PIEZO1

We downloaded TCGA-LIHC RNA-seq data to identify the co-expression partners of PIEZO1. Employing the STRING database (https://cn.string-db.org/), we detected the PPI network for PIEZO1 (22). With Tumor Immune Estimation Resource 2.0 (TIMER2) (http://timer.cistrome.org/), we explored the relationship between PIEZO1 and its co-expression partners in pan-cancer (23). In R, we used the “survival” and “survminer” packages to evaluate the prognostic significance of the co-expression partners of PIEZO1. Finally, GSCALite (https://guolab.wchscu.cn/GSCA/#/) enabled us to explore the signaling pathways related to the co-expression partners of PIEZO1 (24).

### Distribution and expression of PIEZO1 at the single cell

We downloaded the single-cell data from the TISCH database and analyzed the PIEZO1 expression patterns using “MAESTRO” and “Seurat” packages in R language, followed by t-SNE for clustering cells into different groups.

Furthermore, to verify the cell type-specific expression pattern of PIEZO1, we analyzed three publicly available hepatocellular carcinoma scRNA-seq datasets from the GEO database (GSE149614, GSE166635, GSE290925) (25–27). Using the Seurat package for data integration and processing, we specifically isolated hepatocytes based on cell annotation labels with the dplyr package. Subsequently, the filtered hepatocytes were grouped according to their tissue origin (tumor vs. normal tissue), followed by comparison of the average PIEZO1 expression level and the percentage of PIEZO1-positive cells across groups.

### Immunotherapy and drug response of PIEZO1

We downloaded RNA-seq data and corresponding clinical information from the TCGA database and used “ggplot2 “ and “ggpubr “ packages in R language to calculate the Tumor Immune Dysfunction and Exclusion (TIDE) score, evaluating the risk of immune evasion in cancer patients with high or low PIEZO1 expression (28). Additionally, the correlation between PIEZO1 and drug response was analyzed by the BEST database (https://rookieutopia.com/app_direct/BEST/).

### Ethical approval and tissue collection

This study, approved by the ethics committee of the Second Affiliated Hospital of Xi’an Jiaotong University, involved the collection of tissue samples from LIHC and STAD cases, along with adjacent non-tumor tissues. Written informed consent was obtained from all participants. The collected samples were promptly frozen and stored at −80 °C for further analysis.

### Quantitative Real-Time PCR

Total RNA was isolated from patient tissues and cultured cells using the TRIZOL reagent (Invitrogen) and was reversely transcribed into cDNA with the PrimeScript RT reagent Kit (Takara). RT-PCR was performed using the SYBR Premix Ex Taq II Kit (Takara) with primers for PIEZO1 (forward: TTCCTGCTGTACCAGTACCT; reverse: AGGTACAGCCACTTGATGAG) and GAPDH (forward: CGGAGTCAACGGATTTGGTCGTAT; reverse: AGCCTTCTCCATGGTGGTGAAGAC), with GAPDH as the housekeeping gene. The relative levels of target mRNA were normalized to the GAPDH via the 2-ΔΔCT formula.

### Western Blot

Tissue samples were lysed in RIPA buffer (Beyotime, China) to extract proteins, which were subsequently quantified using the BCA assay (Beyotime, China). The protein lysates were then subjected to 8% Sodium Dodecyl Sulfate Polyacrylamide Gel Electrophoresis (SDS-PAGE) and electrotransferred onto a Polyvinylidene Difluoride (PVDF) membrane. For each lane, 40 μg of protein was loaded. The membrane was incubated with primary antibodies specific to PIEZO1 (1:500, Invitrogen, catalog # MA5-32876) and GAPDH (1:2000, CST), overnight at 4 °C. After washing with Tris Buffered Saline Tween (TBST), the membrane was incubated with secondary antibodies for 2 hours at room temperature. Protein bands were visualized using an Enhanced Chemiluminescence (ECL) Western Blot Detection Kit (Millipore). The densitometry of the selected bands was analyzed by Image J software, and PIEZO1 expression levels were normalized to GAPDH, which served as a housekeeping gene on separate gels due to molecular weight differences. All WB experiments included three biological replicates and three technical replicates.

### Cell culture

The human hepatocellular carcinoma cell lines HepG2 and Hep3B, purchased from the Cell Bank of the Chinese Academy of Sciences, were cultured in complete DMEM medium supplemented with 10% Fetal Bovine Serum (FBS) (Gibco, Thermo Fisher Scientific, Inc., USA), 100 U/ml penicillin, and 100 μg/ml streptomycin (Beyotime, Shanghai, China). All cells were maintained at 37°C in a humidified incubator with 5% CO_2_. When cells reached approximately 90% confluence, they were detached using 0.25% trypsin (Beyotime, Shanghai, China) for 1 minute at 37°C and subsequently subcultured or used for subsequent experiments. All cell-based experiments were performed with three technical and biological replicates.

### Cell viability assay

Cells (2×10^3^ cells/well) were seeded into 96-well plates. After adherence, cells were exposed to yoda1 (diluted in DMEM from a 10 mM DMSO stock filtered through 0.22 μm) at concentrations of 0, 10, 50, 100, and 500 μmol/L for 48 h. Then, 10 μL of CCK-8 reagent was added to each well, followed by incubation at 37 °C for 2 h. Optical density (OD450) was measured using a microplate reader (Thermo Fisher Scientific, USA).

### Colony formation assay

Cells (1×10^3^ cells/well) were seeded into 6‐well plates. After adherence, cells were treated with 50 μmol/L yoda1 (prepared as above) or vehicle (DMEM with equivalent DMSO). After 14 days, cells were washed with PBS, fixed in methanol for 15 min, and stained with 1% crystal violet for 15 min. Colonies were counted under a microscope.

### Wound healing assay

The ibidi Culture-Insert 2 Well (ibidi GmbH, Germany, 80209) was placed in a 60-mm Petri dish. Cells were seeded into the left and right chambers of the insert at a density of 5 × 10^5^ cells/well, respectively. After 12 hours of incubation in serum-free medium, the culture insert was carefully removed. yoda1 (50 μmol/L in DMEM) or vehicle was added. Cell migration patterns were documented at 0 h, 8 h, 16 h and 24 h using an inverted microscope.

### Animal studies

In this study, 6-week-old BALB/c nude mice (n = 5 per group) were obtained from the Laboratory Animal Center of Xi’an Jiaotong University. All animal procedures were strictly followed institutional guidelines. Mice were subcutaneously injected with 5×10^5^ H22 cells into the flank. Tumor volume was calculated using the formula: volume = 0.5 × length × width². When tumors reached 100–150 mm³, mice were randomly assigned to receive intraperitoneal injections of PBS (0.1 mL/kg) or yoda1 (4 μg/kg in saline) every 2 days. Tumor growth was monitored daily. After 2 weeks, mice were sacrificed, and tumors were removed for weighing and imaging.

### Statistical analysis

The data analysis and visualization were conducted using R (version 4.3.3) and GraphPad Prism (version 8.0), with the following R packages: TCGAbiolinks (v2.24.3) for data acquisition, limma (v3.52.4) for differential expression analysis, pROC (v1.18.0) for ROC curve analysis, survival (v3.4-0) and survminer (v0.4.9) for survival analysis, and ggplot2 (v3.4.0) for plotting. The Welch one-way ANOVA assessed differences across multiple groups, while the Student t-test compared two groups. Each experiment was repeated three times, with results presented as mean ± Standard Deviation (SD). *p* < 0.05 indicated statistical significance.

## Results

### Expression level of PIEZO1

**Figure 1:**
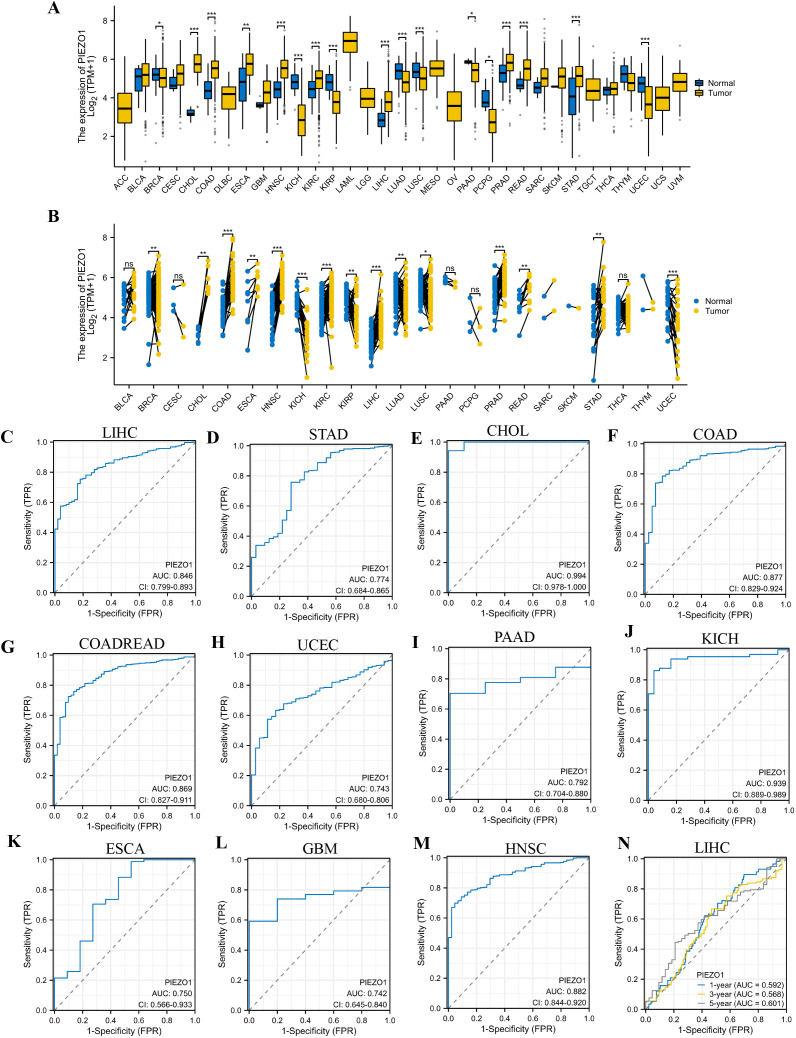
Expression level and diagnostic value of PIEZO1. **(A, B)** PIEZO1 mRNA expression in unpaired and paired cancerous and normal tissues based on the TCGA database. (ns: no significance; *p < 0.05; **p < 0.01; ***p < 0.001). **(C–M)** The diagnostic value of PIEZO1 was determined using ROC curves. **(N)** The predictive efficiency of PIEZO1 was determined using time-dependent ROC curves.

Based on the TCGA database, we first examined PIEZO1 expression in different cancerous and normal tissues, and found PIEZO1 expression was higher in 9 unpaired and paired cancer tissues, including Cholangiocarcinoma (CHOL), Colon Adenocarcinoma (COAD), Esophageal Carcinoma (ESCA), Head and Neck Squamous Cell Carcinoma (HNSC), Kidney Renal Clear Cell Carcinoma (KIRC), Liver Hepatocellular Carcinoma (LIHC), Prostate Adenocarcinoma (PRAD), Rectum Adenocarcinoma (READ), and Stomach Adenocarcinoma (STAD), compared to normal tissues (*p* < 0.05) (**Figures 1A, B**). Further investigation using the HPA dataset revealed PIEZO1 mRNA in multiple cancer cell lines (**Supplementary Figure 1A**). Interestingly, PIEZO1 mRNA expression was mainly detected in SNU-761, Hep 3B2.1-7, and HuH-1 liver cancer cell lines (**Supplementary Figure 1B**).

### Diagnostic value of PIEZO1

Employing Receiver Operating Characteristic (ROC) curves, we calculated the Area Under Curve (AUC) to evaluate the diagnostic value of PIEZO1 across 33 cancers. PIEZO1 demonstrated strong diagnostic accuracy in 16 tumor types, including CHOL (AUC = 0.994, 95% CI: 0.978-1.000), COAD (AUC = 0.877, 95% CI: 0.829-0.924), Colon Adenocarcinoma/Rectum Adenocarcinoma (COADREAD) (AUC = 0.869, 95% CI: 0.827-0.911), Uterine Corpus Endometrial Carcinoma (UCEC) (AUC = 0.743, 95% CI: 0.680-0.806), ESCA (AUC = 0.750, 95% CI: 0.566-0.933), Glioblastoma Multiforme (GBM) (AUC = 0.742, 95% CI: 0.645-0.840), HNSC (AUC = 0.882, 95% CI: 0.844-0.920), Kidney Chromophobe (KICH) (AUC = 0.939, 95% CI: 0.889-0.989), Kidney Renal Papillary Cell Carcinoma (KIRP) (AUC = 0.828, 95% CI: 0.772-0.883), LIHC (AUC = 0.846, 95% CI: 0.799-0.893), Oral Squamous Cell Carcinoma (OSCC) (AUC = 0.905, 95% CI: 0.863-0.947), Pancreatic Adenocarcinoma (PAAD) (AUC = 0.792, 95% CI: 0.704-0.880), Pheochromocytoma and Paraganglioma (PCPG) (AUC = 0.846, 95% CI: 0.705-0.987), PRAD (AUC = 0.744, 95% CI: 0.683-0.806), READ (AUC = 0.829, 95% CI: 0.735-0.924), STAD (AUC = 0.774, 95% CI: 0.684-0.865) (**Figures 1C–M**, **Supplementary Figures 1C–G**). Furthermore, PIEZO1 demonstrated a significant value in predicting the 1-, 3-, and 5-year OS rates of patients with LIHC, as evidenced by the time-dependent ROC analysis (**Figure 1N**). PIEZO1 shows high diagnostic accuracy (AUC > 0.7) in 16 cancer types, supporting its role as a pan-cancer diagnostic biomarker.

### Prognostic value of PIEZO1

**Figure 2:**
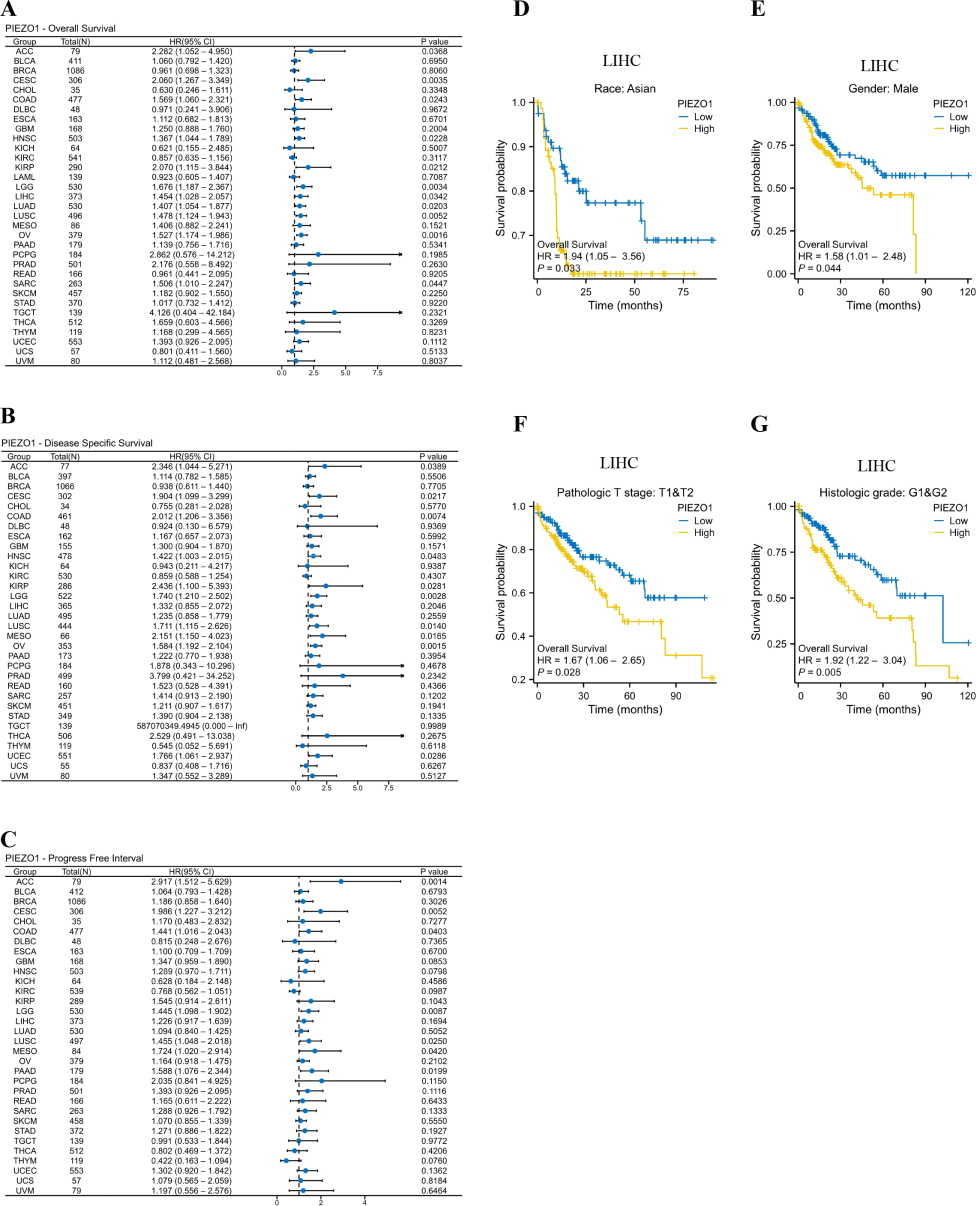
Prognostic value of PIEZO1. **(A–C)** Cox regression analysis was used to detect the correlations between PIEZO1 and OS **(A)**, DSS **(B)**, and PFI **(C)**. **(D–G)** Kaplan-Meier analysis was used to evaluate OS in different LIHC subgroups stratified by high or low PIEZO1 expression.

We investigated the correlation between PIEZO1 expression and OS, DSS, as well as PFI among patients with various cancers using Cox regression analysis and Kaplan-Meier analysis. We found that high PIEZO1 expression had unfavorable OS in 11 cancer types, including Adrenocortical Carcinoma (ACC) (HR = 2.282, 95% CI: 1.052-4.950, *p* = 0.0368), Endocervical Adenocarcinoma (CESC) (HR = 2.060, 95% CI: 1.267-3.349, *p* = 0.0035), COAD (HR = 1.569, 95% CI: 1.060-2.321, *p* = 0.0243), HNSC (HR = 1.367, 95% CI: 1.044-1.789, *p* = 0.0228), KIRP (HR = 2.070, 95% CI: 1.115-3.844, *p* = 0.0212), Low-Grade Glioma (LGG) (HR = 1.676, 95% CI: 1.187-2.367, *p* = 0.0034), LIHC (HR = 1.454, 95% CI: 1.028-2.057, *p* = 0.0342), Lung Adenocarcinoma (LUAD) (HR = 1.407, 95% CI: 1.054-1.877, *p* = 0.0203), Lung Squamous Cell Carcinoma (LUSC) (HR = 1.478, 95% CI: 1.124-1.943, *p* = 0.0052), Ovarian Cancer (OV) (HR = 1.527, 95% CI: 1.174-1.986, *p* = 0.0016), Sarcoma (SARC) (HR = 1.506, 95% CI: 1.010-2.247, *p* = 0.0447) (**Figure 2A**). We further employed a forest plot to visualize results after Benjamini-Hochberg correction, as shown in **Supplementary Figure 2A**. The corresponding Kaplan-Meier curves of OS were presented in **Supplementary Figures 2B–L**.

As for DSS, upregulated PIEZO1 expression was associated with shorter OS in 10 cancer types, including, ACC (HR = 2.346, 95% CI: 1.044-5.271, *p* = 0.0389), CESC (HR = 1.904, 95% CI: 1.099-3.299, *p* = 0.0217), COAD (HR = 2.012, 95% CI: 1.206-3.356, *p* = 0.0074), HNSC (HR = 1.422, 95% CI: 1.003-2.015, *p* = 0.0483), KIRP (HR = 2.436, 95% CI: 1.100-5.393, *p* = 0.0281), LGG (HR = 1.740, 95% CI: 1.210-2.502, *p* = 0.0028), LUSC (HR = 1.711, 95% CI: 1.115-2.626, *p* = 0.0140), MESO (HR = 2.151, 95% CI: 1.150-4.023, *p* = 0.0165), OV (HR = 1.584, 95% CI: 1.192-2.104, *p* = 0.0015), UCEC (HR = 1.766, 95% CI: 1.061-2.937, *p* = 0.0286) (**Figure 2B**). We further employed a forest plot to visualize results after Benjamini-Hochberg correction, as shown in **Supplementary Figure 3A**. The corresponding Kaplan-Meier curves of DSS were presented in **Supplementary Figures 3B–K**.

As for PFI, elevated PIEZO1 expression was associated with poorer OS in 7 cancer types, including, ACC (HR = 2.917, 95% CI: 1.512-5.629, p = 0.0014), CESC (HR = 1.986, 95% CI: 1.227-3.212, p = 0.0052), COAD (HR = 1.441, 95% CI: 1.016-2.043, p = 0.0403), LGG (HR = 1.445, 95% CI: 1.098-1.902, p = 0.0087), LUSC (HR = 1.455, 95% CI: 1.048-2.018, p = 0.0250), MESO (HR = 1.724, 95% CI: 1.020-2.914, p = 0.0420), PAAD (HR = 1.588, 95% CI: 1.076-2.344, p = 0.0199) (**Figure 2C**). We further employed a forest plot to visualize results after Benjamini-Hochberg correction, as shown in **Supplementary Figure 4A**. The corresponding Kaplan-Meier curves of PFI were presented in **Supplementary Figures 4B–H**.

Interestingly, in the LIHC subgroup, patients with Asian, Males, pathologic T stages T1 and T2, and Histologic grades G1 and G2, high PIEZO1 expression correlated with poorer OS compared to low expression (**Figures 2D–G**).

Tumor samples are of high purity among most tumor types in the pan-cancer TCGA database (**Supplementary Figure 5A**). To account for this confounding factor, we adjusted for the ESTIMATE Score in multivariate Cox proportional hazards regression models. The results demonstrated that high PIEZO1 expression remained an independent risk factor for poor prognosis across multiple cancers, significantly shortening OS, DSS, and PFI in patients with various cancer types (HR > 1, *p* < 0.05); (**Supplementary Figures 5B–D**).

Given the established role of age as a significant prognostic factor in cancer, we constructed an age-adjusted multivariate Cox regression model for validation analysis. After adjusting for age, high expression of PIEZO1 remained a significant predictor of prognosis across multiple cancer types. Specifically, in the OS analysis, cancers such as LGG and ACC continued to exhibit significant prognostic associations following age adjustment, further supporting the potential value of PIEZO1 as an independent prognostic biomarker (**Supplementary Figures 5E–G**).

### Relationship between PIEZO1 and clinical features

**Figure 3:**
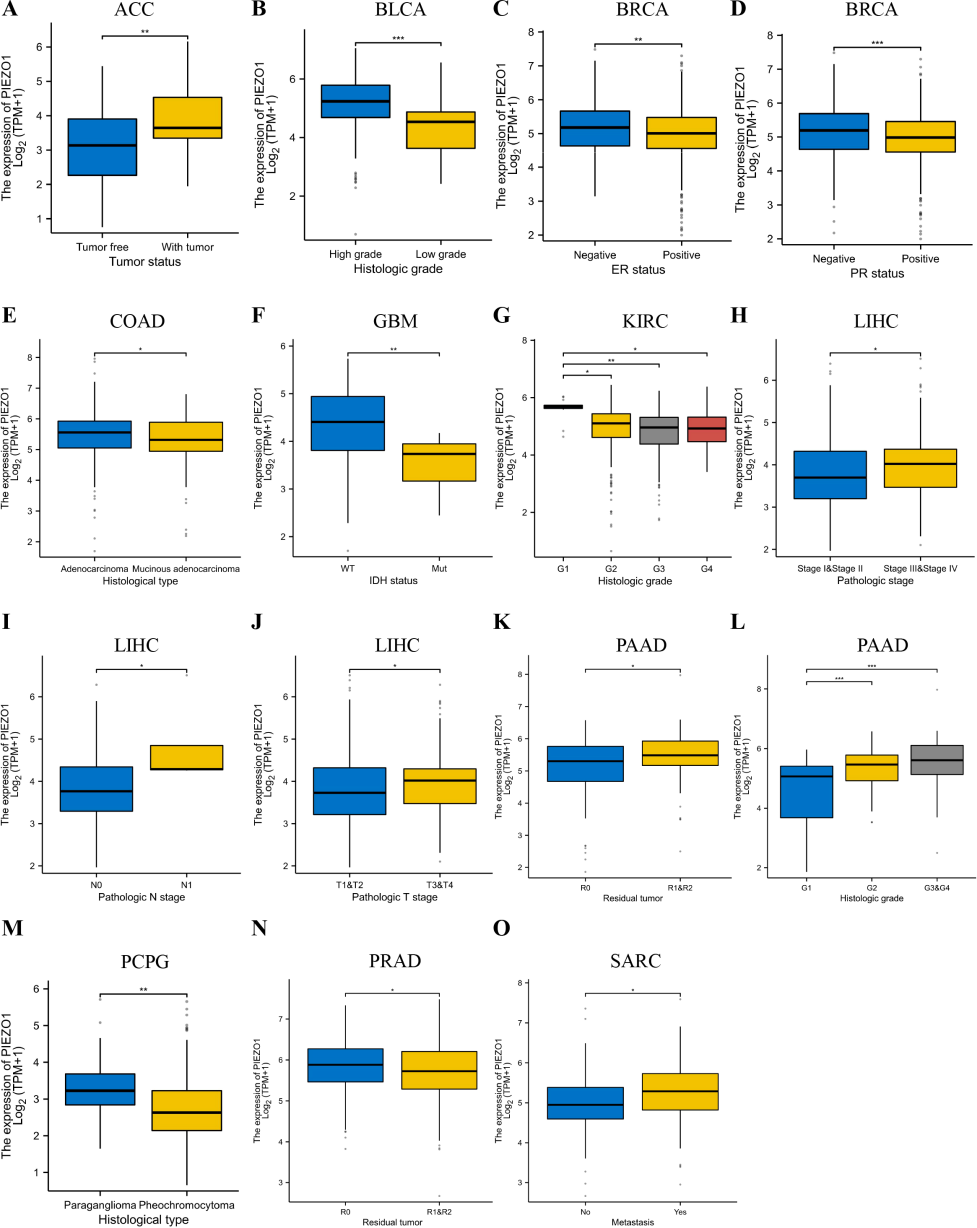
Relationship between PIEZO1 and clinical features. **(A)** The relationship between PIEZO1 and tumor status in ACC. **(B)** The relationship between PIEZO1 and histologic grade in BLCA. **(C)** The relationship between PIEZO1 and ER statuses in BRCA. **(D)** The relationship between PIEZO1 and PR statuses in BRCA. **(E)** The relationship between PIEZO1 and histological type in COAD. **(F)** The relationship between PIEZO1 and IDH status in GBM. **(G)** The relationship between PIEZO1 and histologic grade in KIRC. **(H)** The relationship between PIEZO1 and the pathologic stage in LIHC. **(I)** The relationship between PIEZO1 and pathological N stage in LIHC. **(J)** The relationship between PIEZO1 and pathologic T stage in LIHC. **(K)** The relationship between PIEZO1 and residual tumor in PAAD. **(L)** The relationship between PIEZO1 and histologic grade in PAAD. **(M)** The relationship between PIEZO1 and histological type in PCPG. **(N)** The relationship between PIEZO1 and residual tumor in PRAD. **(O)** The relationship between PIEZO1 and metastasis in SARC. (*p < 0.05; **p < 0.01; ***p < 0.001).

We then detected PIEZO1 association with clinical features across different cancer types. In ACC, tumors exhibited elevated PIEZO1 expression relative to non-tumorous tissues (**Figure 3A**). For BLCA, higher grades were linked to increased PIEZO1 levels (**Figure 3B**). In BRCA, PIEZO1 expression was higher in patients with negative Estrogen Receptor (ER) and Progesterone Receptor (PR) statuses (**Figures 3C, D**). In COAD, adenocarcinoma patients showed heightened PIEZO1 expression (**Figure 3E**). Additionally, in GBM, elevated PIEZO1 expression correlated with the Wild-Type (WT) IDH status (**Figure 3F**). An inverse relationship between PIEZO1 expression and histologic grade was observed in KIRC (**Figure 3G**). Furthermore, in LIHC, the advanced pathologic stage, pathologic N and T stage, were associated with higher PIEZO1 expression (**Figures 3H–J**). PAAD patients with residual tumors classified as R1 and R2 demonstrated increased PIEZO1 levels compared to those with R0, and higher histologic grades were also associated with elevated PIEZO1 expression (**Figures 3K–L**). In PCPG, patients with paraganglioma exhibited higher PIEZO1 expression (**Figure 3M**). Interestingly, PRAD patients with no residual tumor (R0) had higher PIEZO1 expression than those with R1 and R2 (**Figure 3N**). Lastly, in SARC, patients with metastasis displayed increased PIEZO1 expression (**Figure 3O**). Elevated PIEZO1 correlates with advanced tumor stages (e.g., LIHC, PAAD) and metastatic status (SARC), indicating its association with aggressive phenotypes. Furthermore, using the TCGA dataset, we conducted a logistic regression analysis to examine the correlation between PIEZO1 expression levels and clinical features among 374 LIHC patients. As shown in **Table 1**, there was a significant correlation between PIEZO1 expression and pathologic stage (*p* = 0.001).

**Table 1:** PIEZO1 expression correlated with clinical characteristics by logistic regression.

Characteristics	Total (N)	OR (95% CI)	P value
Pathologic T stage (T2&T3&T4 vs. T1)	371	1.460 (0.970 – 2.197)	0.070
Pathologic N stage (N1 vs. N0)	258	73741846.1587 (0.000 – Inf)	0.994
Pathologic M stage (M1 vs. M0)	272	1.030 (0.143 – 7.422)	0.976
Pathologic stage (Stage III&Stage IV vs. Stage I&Stage II)	350	2.285 (1.391 – 3.753)	**0.001**
Tumor status (With tumor vs. Tumor free)	355	1.179 (0.774 – 1.795)	0.443
Gender (Male vs. Female)	374	0.802 (0.520 – 1.239)	0.320
Age (> 60 vs. <= 60)	373	0.851 (0.567 – 1.279)	0.438
BMI (> 25 vs. <= 25)	337	0.654 (0.426 – 1.006)	0.054
Residual tumor (R1&R2 vs. R0)	345	1.723 (0.652 – 4.554)	0.273
Histologic grade (G3&G4 vs. G1&G2)	369	1.399 (0.915 – 2.139)	0.122
AFP(ng/ml) (> 400 vs. <= 400)	280	1.158 (0.664 – 2.017)	0.605
Albumin(g/dl) (>= 3.5 vs. < 3.5)	300	1.378 (0.799 – 2.378)	0.249
Prothrombin time (> 4 vs. <= 4)	297	1.638 (0.994 – 2.700)	0.053
Child-Pugh grade (B&C vs. A)	241	1.719 (0.706 – 4.188)	0.233
Fibrosis ishak score (1/2&3/4&5&6 vs. 0)	215	0.908 (0.517 – 1.595)	0.738
Vascular invasion (Yes vs. No)	318	1.122 (0.707 – 1.782)	0.624
Adjacent hepatic tissue inflammation (Mild&Severe vs. None)	237	1.053 (0.632 – 1.755)	0.841

Bold values indicate statistically significant associations (*p* =0.001) based on logistic regression analysis.

### Mutation characteristics of PIEZO1

**Figure 4:**
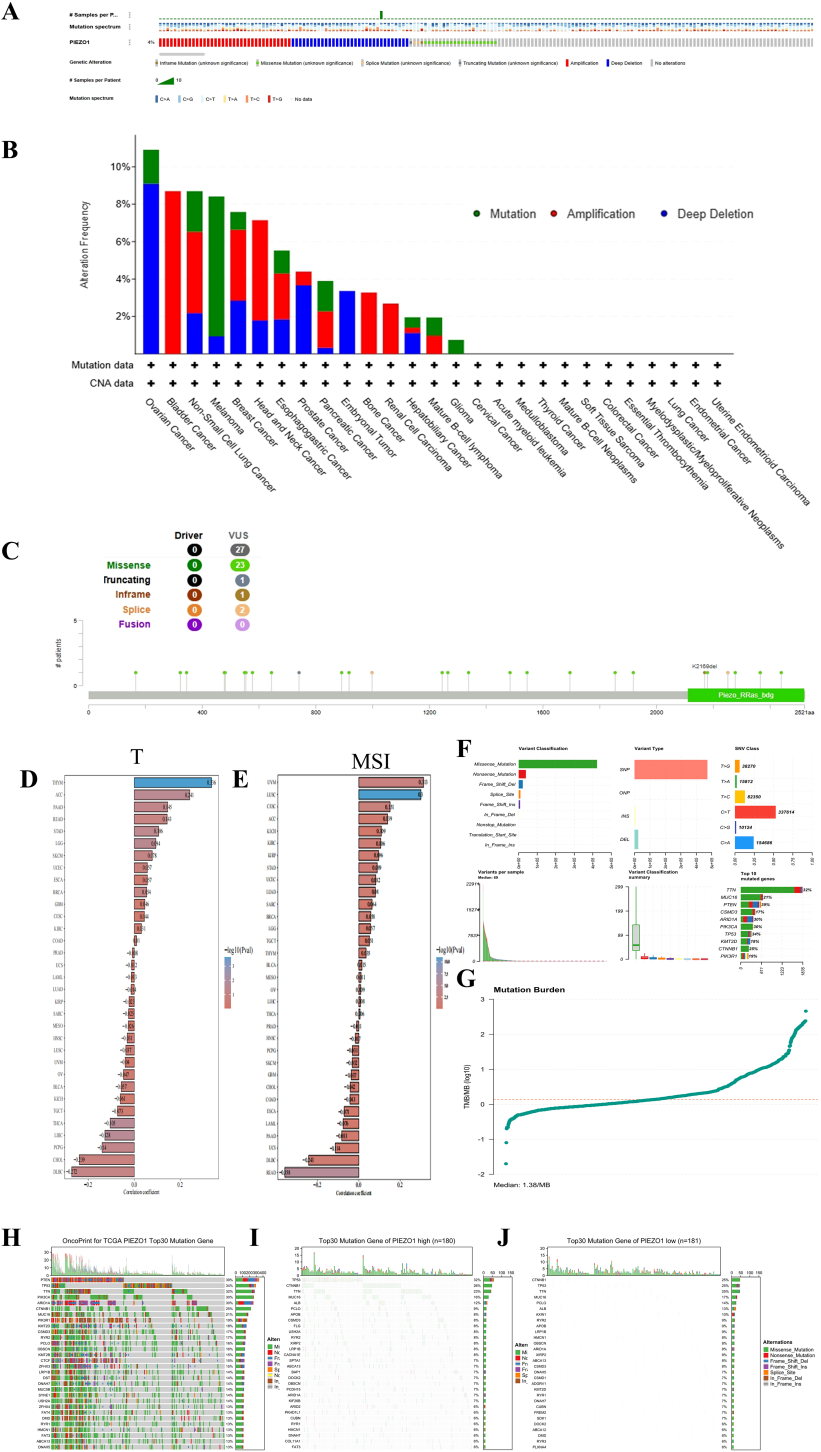
Mutation characteristics of PIEZO1. **(A–C)** Genetic alteration landscape **(A)**, frequencies **(B)**, and sites **(C)** of PIEZO1 across pan-cancer based on cBioportal database. **(D)** The relationship between PIEZO1 and TMB based on the TCGA database. **(E)** The relationship between PIEZO1 and MSI based on the TCGA database. **(F)** Analysis of mutation types in HCC. **(G)** Comparison of TMB in HCC. **(H)** Top 30 frequently mutated genes in all samples. **(I)** Top 30 frequently mutated genes in the PIEZO1 high-expression group. **(J)** Top 30 frequently mutated genes in the PIEZO1 low-expression group.

The cBioportal database indicated that PIEZO1 alterations occurred in 4% (92/2565) of pan-cancer patients, as shown in **Figure 4A**. Further examination revealed mutation, amplification, and deep deletion as the most prevalent forms of PIEZO1 gene modifications across various cancer types. Notably, ovarian cancer had the highest frequency of gene deep deletions, bladder cancer had the highest rate of gene amplifications, and melanoma displayed the highest frequency of gene mutations, as shown in **Figure 4B**. Additionally, analysis of mutation sites revealed missense mutations as the primary form of PIEZO1 gene mutations in pan-cancer, as depicted in **Figure 4C**. Moreover, we found a positive relationship between PIEZO1 and TMB in ACC (*p* = 0.033), LGG (*p* = 0.034), and STAD (*p* = 0.042) (**Figure 4D**), as well as between PIEZO1 and MSI in UVM (*p* = 0.047) and CESC (*p* = 0.008) (**Figure 4E**).

We further investigated the relationship between PIEZO1 expression and genomic alterations in the TCGA-LIHC cohort, and found missense mutation was the predominant variant type in this cohort. Upon stratifying samples based on PIEZO1 expression levels, the high-expression and low-expression groups exhibited distinct somatic mutation profiles. Although TP53 and CTNNB1 were frequently mutated genes in both groups, TP53 mutations occurred at a higher frequency in the PIEZO1 high-expression group, whereas CTNNB1 mutations were more enriched in the PIEZO1 low-expression group. (**Figures 4F–J**)

### Relationship between PIEZO1 and m6A-related genes

Our analysis in LIHC revealed a significant correlation between PIEZO1 expression and m6A regulators (e.g., METTL3, FTO, YTHDF1; **Supplementary Figure 6**), suggesting potential co-regulation of RNA stability or translational efficiency that may amplify PIEZO1’s oncogenic functions.

### The GO and GSEA analysis

**Figure 5:**
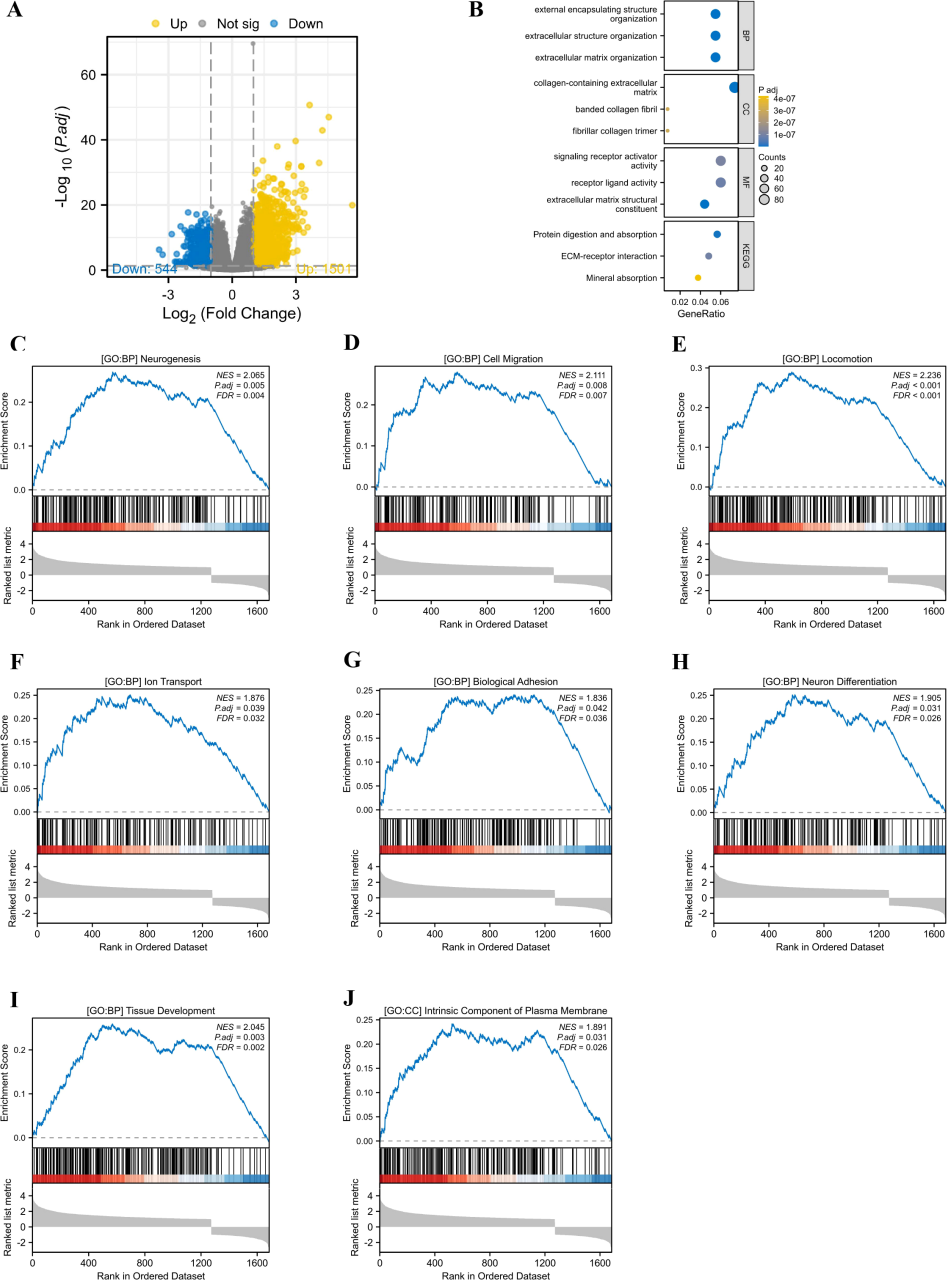
The GO and GSEA analysis. **(A)** The differentially expressed genes between high and low PIEZO1 expression cohorts in TCGA-LIHC patients. **(B)** The GO analysis identified the functions of PIEZO1-associated differentially expressed genes. **(C–J)** The GSEA analysis revealed the biological processes and cellular components of PIEZO1.

Stratified by median PIEZO1 mRNA expression levels from the TCGA database, LIHC patients were categorized into high- and low-expression groups. Our analysis identified 2,0459 differentially expressed genes (1,501 upregulated and 544 downregulated) between these groups, which were then subjected to GO and GSEA (**Figure 5A**).

The GO analysis indicated that the primary functions of PIEZO1-associated differentially expressed genes were focused on external encapsulating structure organization, extracellular structure organization, extracellular matrix organization, collagen-containing extracellular matrix, banded collagen fibril, fibrillar collagen trimer, signaling receptor activator activity, receptor ligand activity, extracellular matrix structural constituent, protein digestion and absorption, ECM-receptor interaction, and mineral absorption (**Figure 5B**).

The GSEA analysis revealed that elevated PIEZO1 expression was significantly correlated with a range of biological processes and cellular components, including neurogenesis (NES = 2.065, P.adj = 0.005) (**Figure 5C**), cell migration (NES = 2.111, P.adj = 0.008) (**Figure 5D**), locomotion (NES = 2.236, P.adj < 0.001) (**Figure 5E**), lon transport (NES = 1.876, P.adj = 0.039) (**Figure 5F**), biological adhesion (NES = 1.836, P.adj = 0.042) (**Figure 5G**), neuron differentiation (NES = 1.905, P.adj = 0.031) (**Figure 5H**), tissue development (NES = 2.045, P.adj = 0.003) (**Figure 5I**), and intrinsic component of plasma membrane (NES = 1.891, P.adj = 0.031) (**Figure 5J**).

### The co-expression partners of PIEZO1

**Figure 6:**
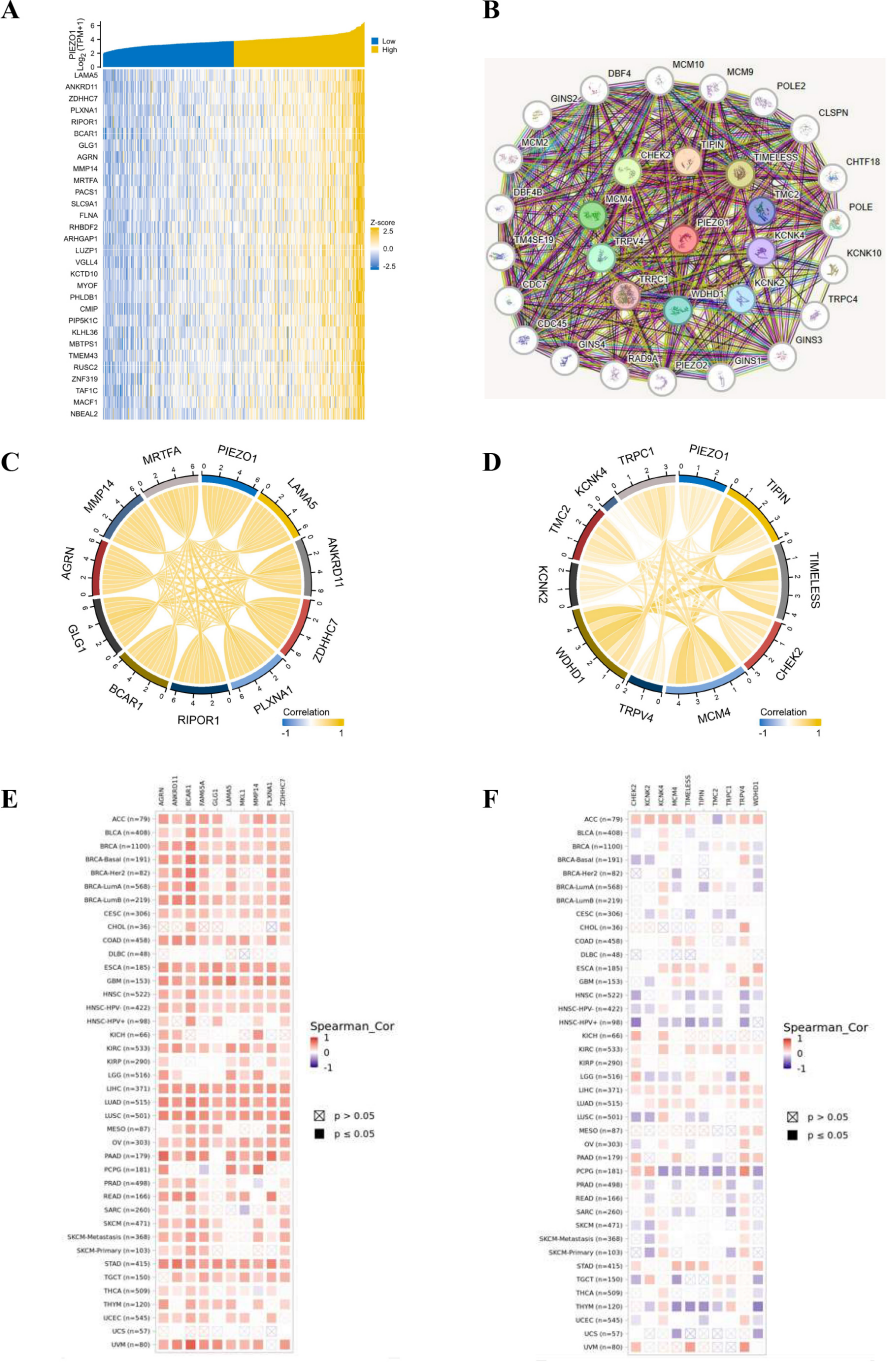
The co-expression partners of PIEZO1. **(A)** The heat map showed the top 30 genes related to PIEZO1 in LIHC based on the TCGA database. **(B)** The network diagram showed the top 30 proteins related to PIEZO1 based on the STRING tool. **(C, D)** The chord diagram showed the correlations between PIEZO1 and the top 10 genes **(C)** and proteins **(D)**. **(E, F)** The relationship between PIEZO1 and its co-expressed partners in pan-cancer based on the TIMER2 database.

Utilizing the TCGA database, we generated a heat map highlighting the top 30 genes related to PIEZO1 in LIHC (**Figure 6A**). Employing the STRING tool, we pinpointed the top 30 proteins related to PIEZO1 (**Figure 6B**). A chord diagram further showed the positive correlations between PIEZO1 and the top 10 genes (MRTFA, MMP14, AGRN, GLG1, BCAR1, PIPOR1, PLXNA1, ZDHHC7, ANKRD11 and LAMA5) or proteins (TRPC1, KCNK4, TMC2, KCNK2, WDHD1, TRPV4, MCM4, CHEK2, TIMELESS and TIPIN), as visualized in **Figures 6C, D**. Additionally, the TIMER2 database further revealed the significant link between PIEZO1 and its co-expressed partners in pan-cancer, as detailed in **Figures 6E, F**. Among co-expressed partners, KM survival curves demonstrated that high MMP14, AGRN, GLG1, BCAR1, PIPOR1, PLXNA1, ZDHHC7, TRPC1, WDHD1, CHEK2, TIMELESS, and TIPIN expression were correlated with poorer OS in LIHC (**Supplementary Figures 7A–L**). Further analysis of PIEZO1 and its co-expressed partners revealed their contribution to LIHC progression via regulating the cell cycle and EMT (**Supplementary Figure 7M**).

### Association of PIEZO1 and TIME

**Figure 7:**
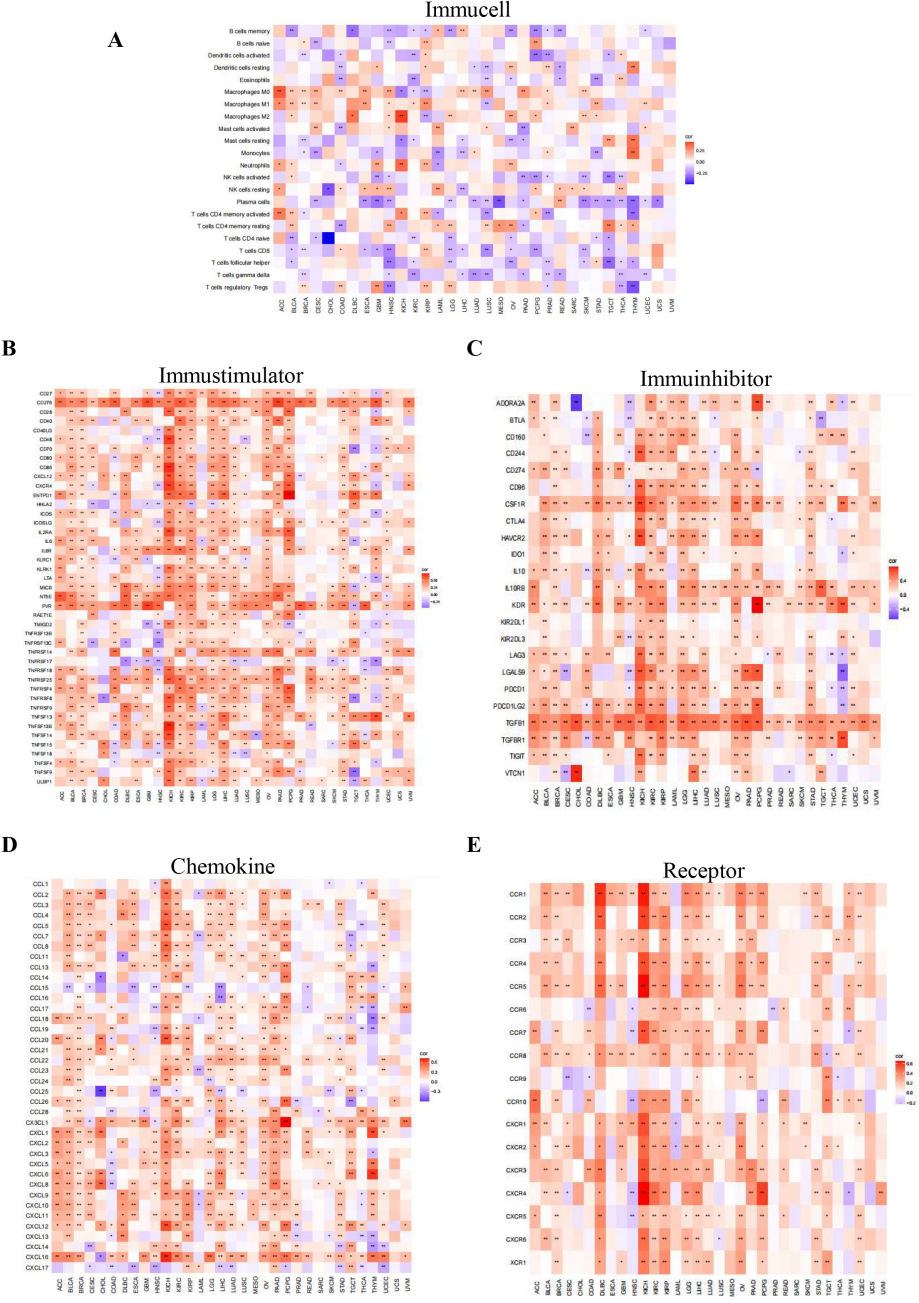
Association of PIEZO1 and TIME. **(A–E)** The relationship between PIEZO1 and immune cells **(A)**, immunostimulators **(B)**, immune inhibitors **(C)**, chemokines **(D)**, and receptors **(E)**. Red indicates positive correlations, while blue signifies negative ones.

Using Spearman correlation analysis, we detected the correlations between PIEZO1 and the components of the TIME, including immune cells, stimulators, inhibitors, chemokines, and receptors. Our analysis uncovered a negative association between PIEZO1 and the majority of immune cells, suggesting a potential role in suppressing the immune response (**Figure 7A**). Additionally, PIEZO1 exhibited a positive correlation with immune stimulators, inhibitors, chemokines, and receptors, indicating its potential to enhance immune signaling pathways (**Figures 7B–E**). These findings highlighted the contrasting role of PIEZO1 within the cancer immune environment.

### Distribution and expression of PIEZO1 at the single cell

Based on the TISCH2 database, we investigated the distribution and expression of PIEZO1 at the single cell in LIHC (**Supplementary Figure 8A**). The LIHC GSE140228 Smartseq2 revealed PIEZO1 expression in Mono. Macro, Tprolif, CD8Tex cells, and other cells, where Mono. Macro cells exhibit the highest levels (**Supplementary Figures 8B, C**). The GSE166635 dataset indicated PIEZO1 expression in Endothelial, Tprolif, DC, and other cells, with Endothelial showing the highest expression (**Supplementary Figures 8D, E**). Similarly, the LIHC GSE98638 highlighted PIEZO1 expression in Treg, Tprolif, CD4Tcom, and other cells, with Treg cells displaying the highest expression (**Supplementary Figures 8F, G**).

Additionally, we analyzed the scRNA-seq data from LIHC. Within the specifically isolated hepatocyte populations, malignant hepatocytes derived from tumor tissues exhibited a significantly higher average expression level of PIEZO1 compared to normal hepatocytes derived from adjacent normal tissues. Furthermore, the proportion of PIEZO1-positive cells was significantly elevated in malignant hepatocytes relative to normal hepatocytes (**Supplementary Figure 9**). These findings provide direct evidence supporting the notion that PIEZO1 overexpression is an intrinsic characteristic of tumor cells.

### The function of PIEZO1 in cancer immunotherapy and drug response

**Figure 8:**
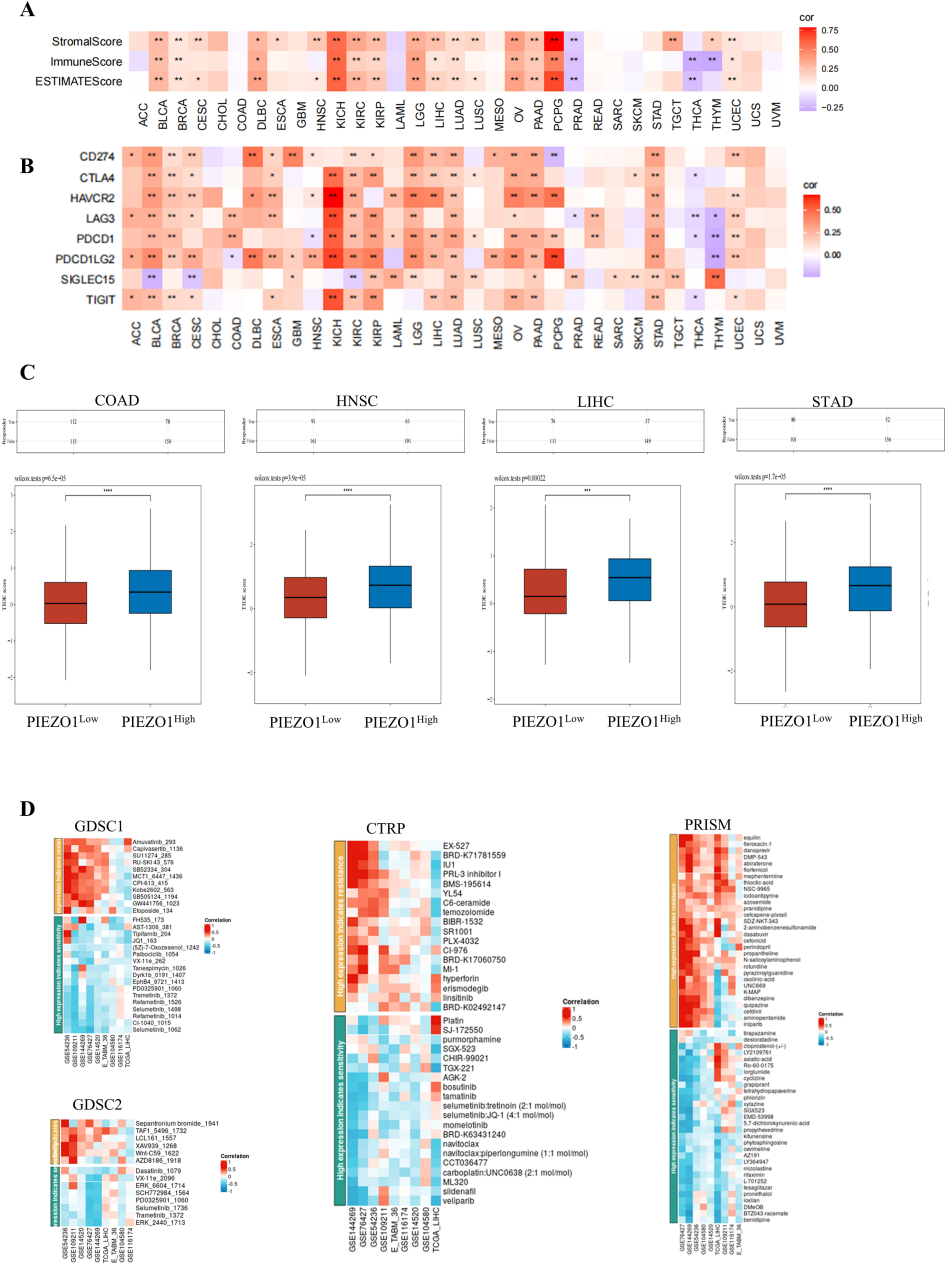
The function of PIEZO1 in cancer immunotherapy and drug response. **(A)** The association of PIEZO1 with StromalScore, ImmuneScore, and ESTIMATEScore in pan-cancer. **(B)** The relationships between PIEZO1 and immune checkpoints in pan-cancer. **(C)** TIDE scores in COCA, HNSC, LIHC, and STAD patients stratified by high or low PIEZO1 expression. **(D)** The function of PIEZO1 in drug response using the BEST database. *p < 0.05; **p < 0.01; ***p < 0.001; ****p < 0.0001.

Our study revealed that PIEZO1 exhibited positive association with StromalScore, ImmuneScore, and ESTIMATEScore in pan-cancer, highlighting its connection to both stromal and immune cells within the immune microenvironment (**Figure 8A**). Furthermore, PIEZO1 showed positive relationships with multiple immune checkpoints, including CD274, CTLA4, HAVCR2, LAG3, PDCD1, PDCD1LG2, SIGLEC15, and TIGIT in pan-cancer (**Figure 8B**). Additionally, we found that the high PIEZO1 expression group had a higher TIDE score across COCA, HNSC, LIHC, and STAD, implying a correlation between elevated PIEZO1 levels and an increased propensity for immune dysfunction and rejection (**Figure 8C**).

Lastly, we found relationships between PIEZO1 expression and drug sensitivity in LIHC across various databases (**Figure 8D**). Specifically, higher PIEZO1 expression in GSE144269 was associated with increased sensitivity to GW441756_1023 (r = 0.697), while lower PIEZO1 expression in GSE76427 was associated with increased sensitivity to Selumetinib_1062 (r = -0.601) within GDSC1 datasets. And, higher PIEZO1 expression in GSE54236 was associated with increased sensitivity to Sepantronium bromide_1941 (r = 0.624), while lower PIEZO1 expression in GSE76427was associated with increased sensitivity to ERK_6604_1714 (r = -0.548) within GDSC2 datasets. Moreover, higher PIEZO1 expression in GSE144269 was associated with increased sensitivity to EX-527 (r = 0.702), while lower PIEZO1 expression in TCGA_LIHC was associated with increased sensitivity to TGX-221 (r = -0.657) within CTRP datasets. In addition, higher PIEZO1 expression in GSE76427 was associated with increased sensitivity to aminopentamide (r = 0.670), while lower PIEZO1 expression in GSE144269 was associated with increased sensitivity to BTZ043-racemate (r = -0.780) within PRISM datasets.

### The expression level of PIEZO1 in LIHC and STAD

**Figure 9:**
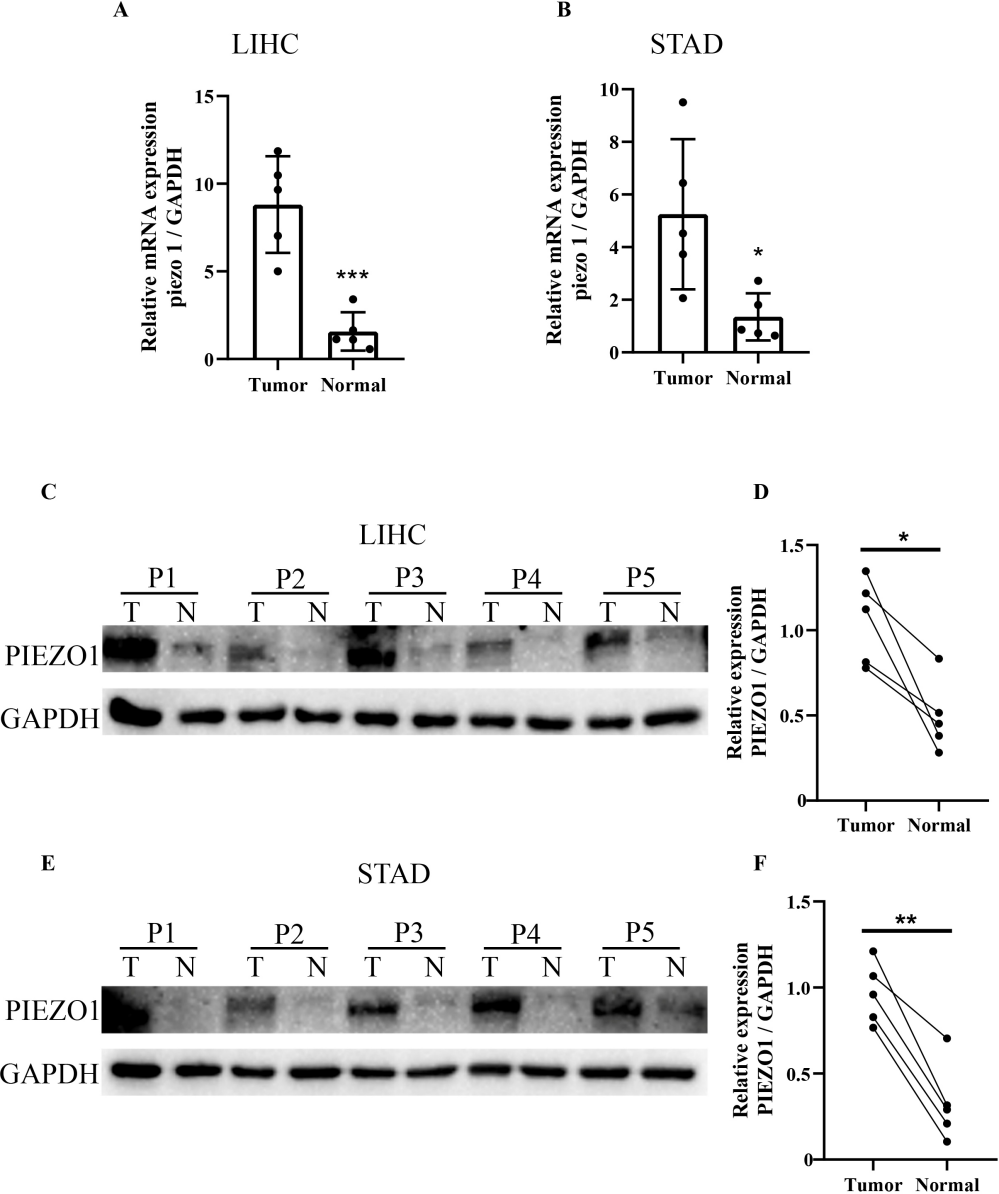
qRT-PCR and WB analysis of PIEZO1 expression in tumor tissues. **(A, B)** qRT-PCR analysis of PIEZO1 expression in LIHC and STAD tissues. Data are presented as mean ± SD of three technical replicates per sample. **(C–F)** WB analysis of PIEZO1 expression in LIHC and STAD tissues. *p < 0.05; **p < 0.01; ***p < 0.001.

We examined PIEZO1 expression in five paired LIHC and STAD specimens, as well as their matched adjacent tissues. Quantitative PCR showed PIEZO1 mRNA upregulation in both LIHC and STAD tissues relative to matched adjacent tissues (**Figures 9A, B**). Western blot analysis further confirmed PIEZO1 protein overexpression in LIHC (**Figures 9C, D**) and STAD (**Figures 9E, F**) tumor tissues. These results indicated PIEZO1’s potential role in the tumorigenesis of both LIHC and STAD.

### The impact of PIEZO1 on cancer cell viability and metastasis *in vitro*

**Figure 10:**
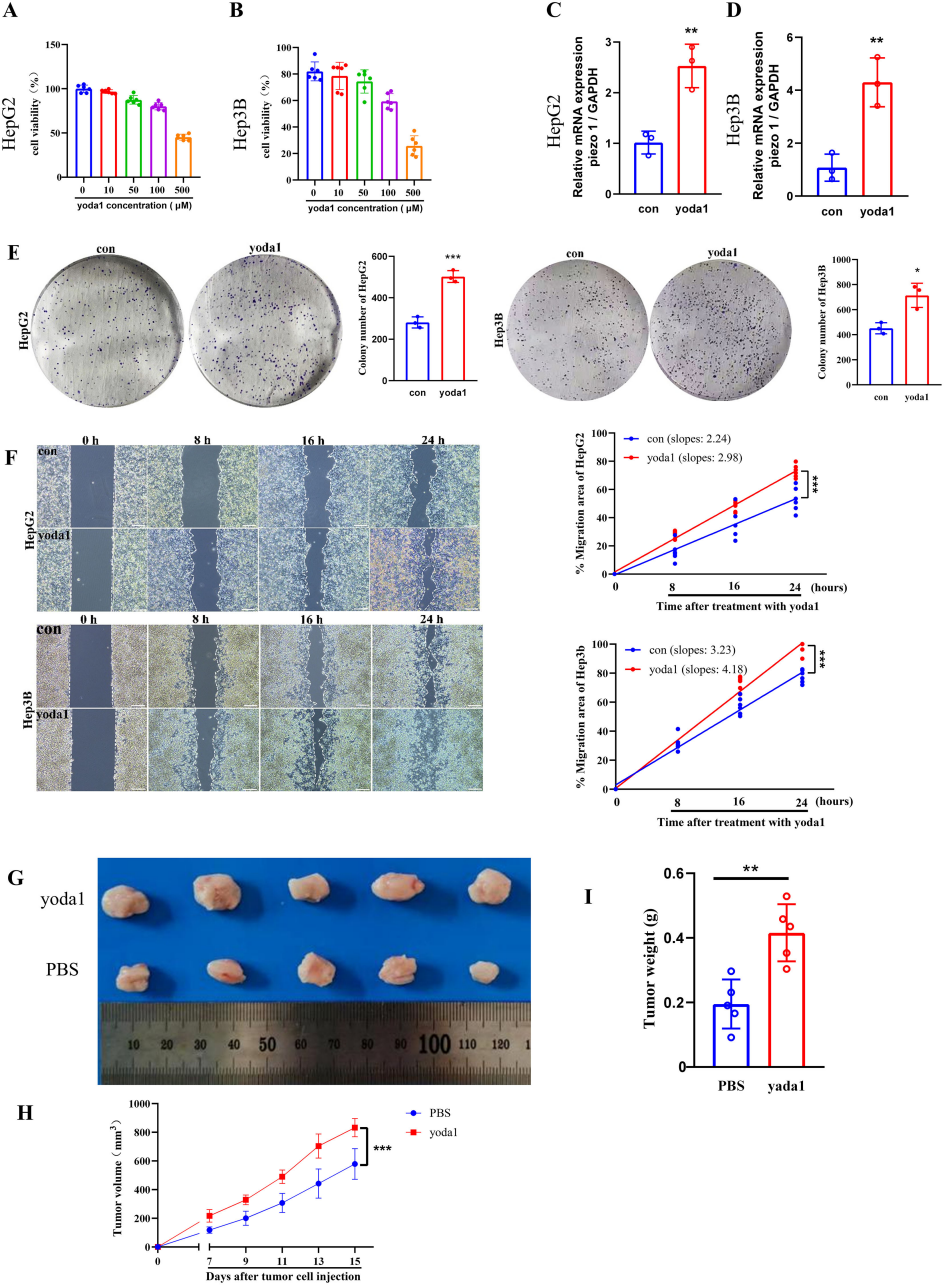
Piezo1 promotes liver cancer cell proliferation and metastasis *in vitro* and vivo. **(A, B)** CCK-8 assay results of liver cancer cells exposed to varying yoda1 concentrations. **(C, D)** qRT-PCR analysis of PIEZO1 expression in liver cancer cells treated with 50 μM yoda1 versus controls. **(E)** Representative colony formation assay images and statistics for liver cancer cells treated with 50 μM yoda1 or PBS. **(F)** Representative wound healing assay images and statistics for liver cancer cells treated with 50 μM yoda1 or PBS, scale bar = 50 μm. **(G)** Xenograft tumor images in yoda1 and control groups. **(H, I)** The volume and weight of tumors were analyzed in the yoda1 and control groups. (*p < 0.05; **p < 0.01; ***p < 0.001).

To investigate PIEZO1’s influence on cancer cell viability, HepG2 and Hep3B cells were exposed to yoda1, a PIEZO1 activator. As yoda1 doses increased, cell viability was inhibited, establishing 50 μM as the optimal concentration for subsequent experiments (**Figures 10A, B**). qPCR analysis demonstrated an upregulation of PIEZO1 expression in yoda1-stimulated HepG2 and Hep3B cells (**Figures 10C, D**). Notably, while acute yodal treatment (48 h) suppressed viability, it induced a migration-proliferation switch that significantly enhanced clonogenic capacity in long-term culture (14 days) (**Figure 10E**). These findings suggest that while yoda1 acutely suppresses cell viability, it may also promote colony formation through alternative mechanisms. Wound healing assays further revealed accelerated wound closure in yoda1-stimulated HepG2 and Hep3B cells compared to controls, indicating PIEZO1 activation significantly accelerates cancer cell migration, directly demonstrating its pro-metastatic role (**Figure 10F**).

### The impact of PIEZO1 on tumor growth *in vivo*

To examine PIEZO1’s effect on tumor growth *in vivo*, H22 cells were injected subcutaneously into the flanks of mice, followed by treatment with either PBS or yoda1. Tumors in the yoda1-treated cohort displayed larger size, volume, and weight compared to those in the PBS-treated cohort (**Figures 10G-I**). These results indicated the role of PIEZO1 in promoting tumor growth *in vivo*, highlighting its potential as a therapeutic target.

## Discussion

We comprehensively analyzed PIEZO1 expression and its functional implications by various databases. Firstly, we compared PIEZO1 expression between normal and cancerous tissues, and found a higher PIEZO1 expression in CHOL, COAD, ESCA, HNSC, KIRC, LIHC, PRAD, READ, and STAD, indicative of its potential as an oncogene. Secondly, we used ROC curves to assess the diagnostic value of PIEZO1, and found that PIEZO1 showed good diagnostic value in 16 tumor types with AUC > 0.700. Thirdly, Cox regression analysis and Kaplan-Meier analysis confirmed a strong correlation between elevated PIEZO1 expression and adverse survival outcomes in multiple cancers. Collectively, our results position PIEZO1 as a promising biomarker for both diagnosis and prognosis in pan-cancer.

Subsequently, we explored the correlation between PIEZO1 expression and clinical characteristics. Collectively, our pan-cancer analysis revealed that elevated PIEZO1 expression correlates with features of advanced and aggressive disease across multiple malignancies, including higher tumor grade (BLCA, PAAD), advanced stage (LIHC), metastatic potential (SARC), and hormone receptor negativity (BRCA). These associations align with poor clinical outcomes, positioning PIEZO1 as a promising biomarker for tumor aggressiveness and prognosis. These features are strongly correlated with tumor aggressiveness, increased risk of recurrence, and poor clinical outcomes. As such, elevated PIEZO1 expression may emerge as a promising prognostic biomarker in cancer management.

Interestingly, PIEZO1 expression was significantly downregulated in KICH compared to normal tissue, contrasting with its oncogenic role observed in other cancers. This context-dependent behavior parallels the reported function of other mechanosensitive proteins, such as TRPV4, which promotes breast cancer metastasis but inhibits glioma progression (29, 30). We hypothesize that KICH’s unique extracellular matrix (ECM) architecture (e.g., low stiffness) or characteristic driver mutations (e.g., loss) may attenuate PIEZO1-dependent mechanosignaling, potentially contributing to its less aggressive clinical course (31). Further studies are needed to determine whether PIEZO1 silencing in KICH represents a passive bystander effect or an active tumor-suppressive adaptation.

Our GO and GSEA analysis revealed that PIEZO1 may be crucial in controlling ECM-receptor interaction, cell migration, and ion transport. The ECM-receptor interaction pathways play a crucial role in tumor progression. Co-expression analysis further identified MMP14, AGRN, and LAMA5 as key partners of PIEZO1, suggesting its direct role in ECM remodeling. Mechanistically, PIEZO1 may orchestrate ECM-receptor interactions through: Transcriptional regulation, Ca²^+^-dependent signaling, and Structural coordination. Transcriptomic profiling has revealed the enrichment of ECM-receptor signaling in breast cancer, highlighting its significant role in tumor advancement (32). Additionally, studies have shown that SNHG16 drives the progression of HCC by activating the ECM-receptor interaction pathway (33). Hence, PIEZO1 may contribute to cancer progression by regulating the ECM-receptor signaling pathway. Our findings highlight PIEZO1’s critical role in cancer cell migration, consistent with prior studies. For example, Piezo1 activation via its agonist yoda1 enhances pancreatic stellate cells (PSCs) migration in both 2D and 3D environments in Pancreatic ductal adenocarcinoma (PDAC) (34). And, PIEZO1 drives colon cancer cell growth, migration, and metastasis (35). Furthermore, PIEZO1 acts as a novel trefoil factor family 1 binding protein, enhancing gastric cancer cell movement *in vitro* (36). Ion transporters play a crucial role in cancer progression by regulating cell proliferation, inducing epithelial-mesenchymal transition (EMT), and promoting cell motility and metastasis (37–39). We thus hypothesize that PIEZO1 may modulate the malignant behavior of tumor cells via ion transporters.

In the LIHC GSE98638 dataset, PIEZO1 was found to be most highly expressed in Tregs at the single cell level. Tregs, a specialized subset of CD4^+^ T cells, frequently accumulate and overactivate in carcinomas, contributing to immunosuppression and tumor immune escape (40–42). Moreover, elevated proportions of Tregs are usually correlated with unfavorable outcomes in various cancer types (43–45). The elevated PIEZO1 expression in Tregs may promote tumor immune escape and progression by modulating Treg function within the tumor microenvironment.

Notably, our study identified significant correlations between PIEZO1 and m6A regulators (e.g., METTL3, FTO, IGF2BP1) in LIHC. m6A modification, the most abundant post-transcriptional RNA modification in eukaryotes, governs key oncogenic processes by modulating RNA decay, splicing, and translation efficiency. For instance, METTL3 promotes translation of oncogenes (e.g., EGFR, MYC) via m6A deposition. FTO enhances tumor progression by demethylating m6A on proliferation-related transcripts. The co-expression pattern observed here implies that m6A machinery may stabilize PIEZO1 mRNA or enhance its translation, thereby potentiating its roles in ECM remodeling and metastasis. This synergy aligns with recent studies showing that m6A modifications regulate mechanosensitive pathways in tumors. Targeting the PIEZO1-m6A axis (e.g., using m6A inhibitor STM2457) could thus represent a novel combinatorial strategy against PIEZO1-driven cancers.

We found that PIEZO1 expression significantly correlates with stromal score and immune score for various malignancies, suggesting its dual regulatory role in the TME. Cancer-associated fibroblasts (CAFs), the primary stromal cells, drive cancer progression and influence treatment response and are associated with poor patient outcomes (46, 47). For instance, H. pylori‐NF‐κB activates the PIEZO1-YAP1-CTGF pathway, enhancing CAF infiltration to remodel the GC microenvironment (48). Additionally, Piezo1 supports optimal T-cell activation during tumor challenges (49). Overall, the complex role of PIEZO1 in the TME highlights the need to explore TME regulatory mechanisms and offers insights for immunotherapy research.

Our research shows that PIEZO1 expression is positively correlated with key immune checkpoints, including CD274 (PD-L1), CTLA4, LAG3, PDCD1 (PD-1), PDCD1LG2 (PD-L2), and TIGIT across various cancer types. PD-L1 is highly expressed in many cancers, enabling tumors to evade T-cell immunity via PD-L1/PD-1 signaling (50). CTLA4 is crucial for suppressing activated T lymphocyte immune responses (51). LAG3 and TIGIT contribute to immune evasion and T cell exhaustion (52). PD-1 binding to PD-L1/PD-L2 reduces cytokine production and T-cell proliferation (53). Thus, PIEZO1’s positive correlation with these checkpoints suggests it may create an immunosuppressive TME, leading to rapid tumor progression.

Our *in vitro* and *in vivo* experiments highlight PIEZO1’s role in promoting LIHC progression. In colony formation assays, liver cancer cells stimulated with PIEZO1 activator (yoda1) exhibited enhanced proliferative capacity. Similarly, wound healing assays demonstrated faster wound closure in these cells. Consistent with these *in vitro* findings, tumors derived from the yoda1-treated group exhibited larger size, volume, and weight in comparison to those from the PBS-treated group in the *in vivo* experiments. Notably, while 50 μM yoda1 did not significantly alter the overall viability of HCC cells within 48 hours, it markedly enhanced their migratory capacity and long-term clonogenic potential. This suggests that the pro-growth effects of Piezo1 activation on HCC cells are time-dependent: acute activation primarily drives migration, whereas chronic sustained activation significantly promotes proliferation and survival. Such dynamic effects were integrally recapitulated *in vivo*, where prolonged yoda1 stimulation ultimately led to a pronounced acceleration of tumor growth. These findings reflect a multistage pro-tumorigenic process wherein Piezo1, in response to persistent mechanical stimuli within the tumor microenvironment, progressively drives invasion and proliferation. Collectively, these results provide compelling evidence that PIEZO1 acts as a facilitator of tumor progression in LIHC.

It is important to acknowledge the limitations of our study. First, data from public databases may have heterogeneity due to different sample collection and sequencing methods. Second, while we confirmed PIEZO1’s role, its downstream effectors need further validation using overexpression or knockout models.

Our focus on LIHC and STAD models for PIEZO1 expression analysis was driven by their dual significance as leading causes of cancer-related mortality in China with high incidence and poor prognosis, coupled with their development within mechanoactive microenvironments (e.g., hepatic sinusoidal shear stress, gastric peristalsis) where PIEZO1’s mechanosensory function may prove pivotal. Future functional validation across diverse malignancies will strengthen conclusion generalizability.

There are some advantages to our study. This first pan-cancer analysis reveals PIEZO1’s differential expression across malignancies and its diagnostic/prognostic biomarker potential. Secondly, PIEZO1 genomic alterations were present in 4% (92/2565) of pan-cancer patients and significantly correlated with m6A-related genes. Thirdly, PIEZO1 may drive tumor progression through ECM-receptor interactions, cell migration, and ion transport. Fourth, PIEZO1 shows positive associations with tumor microenvironment scores (Stromal/Immune/ESTIMATE) and immune checkpoint markers (CD274, CTLA4, LAG3, PDCD1, PDCD1LG2). Fifth, qRT-PCR and WB validated PIEZO1 overexpression in LIHC and STAD tumor specimens. Lastly, *in vitro* and *in vivo* experiments highlighted the role of PIEZO1 in promoting the progression of LIHC. Collectively, our results establish PIEZO1’s oncogenic role and therapeutic potential across cancers.

## Data Availability

The original contributions presented in the study are included in the article/**Supplementary Files**, further inquiries can be directed to the corresponding author/s.

## Supplementary material

Supplementary Figure 1 PIEZO1 expression patterns and diagnostic performance across cancer types. **(A)** PIEZO1 mRNA expression across various cancer cell lines based on the HPA database. **(B)** PIEZO1 mRNA expression across various liver cancer cell lines based on the HPA database. **(C–G)** The diagnostic value of PIEZO1 was determined using ROC curves.

Supplementary Figure 2 Correlations between PIEZO1 and OS. **(A)** Benjamini-Hochberg correction Cox regression analysis was used to detect the correlations between PIEZO1 and OS. **(B–L)** Kaplan-Meier analysis were used to detect the correlations between PIEZO1 and OS.

Supplementary Figure 3 Correlations between PIEZO1 and DSS. **(A)** Benjamini-Hochberg correction Cox regression analysis was used to detect the correlations between PIEZO1 and DSS. **(B–K)** Kaplan-Meier analysis were used to detect the correlations between PIEZO1 and DSS.

Supplementary Figure 4 Correlations between PIEZO1 and PFI. **(A)** Benjamini-Hochberg correction Cox regression analysis was used to detect the correlations between PIEZO1 and PFI. **(B–H)** Kaplan-Meier analysis were used to detect the correlations between PIEZO1 and PFI.

Supplementary Figure 5 Analyses of PIEZO1 purity and, ultifactorial survival in LIHC. **(A)** Radar plot illustrating the association between PIEZO1 expression and tumor microenvironment scores in pan-cancer cohorts. The plot compares StromalScore, ImmuneScore, and ESTIMATEScore between PIEZO1 high-expression group (red line) and low-expression group (blue line). **(B–D)** Prognostic analysis of PIEZO1 expression for OS **(B)**, DSS **(C)**, PFI **(D)**. The forest plot displays HRs of PIEZO1 expression from multivariate Cox regression models adjusted by ESTIMATEScore. **(E–G)** Prognostic analysis of PIEZO1 expression for OS **(E)**, DSS**(F)**, PFI **(G)**. The forest plot displays HRs of PIEZO1 expression from multivariate Cox regression models adjusted by Age.

Supplementary Figure 6 Relationship between PIEZO1 and m6A-related genes. The correlation between PIEZO1 expression and the majority of m6A-related genes in LIHC.

Supplementary Figure 7 The survival and pathway analysis of co-expression partners of PIEZO1. **(A–L)** Kaplan-Meier analysis was used to detect the correlations between co-expression partners of PIEZO1 and OS in LIHC. **(M)** The pathway analysis of PIEZO1 and its co-expressed partners in LIHC using the GSCALite tool

Supplementary Figure 8 Distribution and expression of PIEZO1 at the single cell. **(A)** Distribution and expression of PIEZO1 at the single cell in different LIHC databases using the TISCH2 tool. **(B–G)** Distribution and expression of PIEZO1 at the single cell in LIHC GSE140228 Smartseq2 **(B, C)**, LIHC GSE166635 **(D, E)**, and LIHC GSE98638 **(F, G)**.

Supplementary Figure 9 Expression patterns of PIEZO1 in different cell types.
