# Supplementary figures and images for: Pan-cancer analysis of PIEZO1: a promising biomarker for diagnosis, prognosis, and targeted therapies
Source: Front Immunol. 2025 Sep 4;16:1625734. doi: 10.3389/fimmu.2025.1625734 (PMC12443790; doi:10.3389/fimmu.2025.1625734)

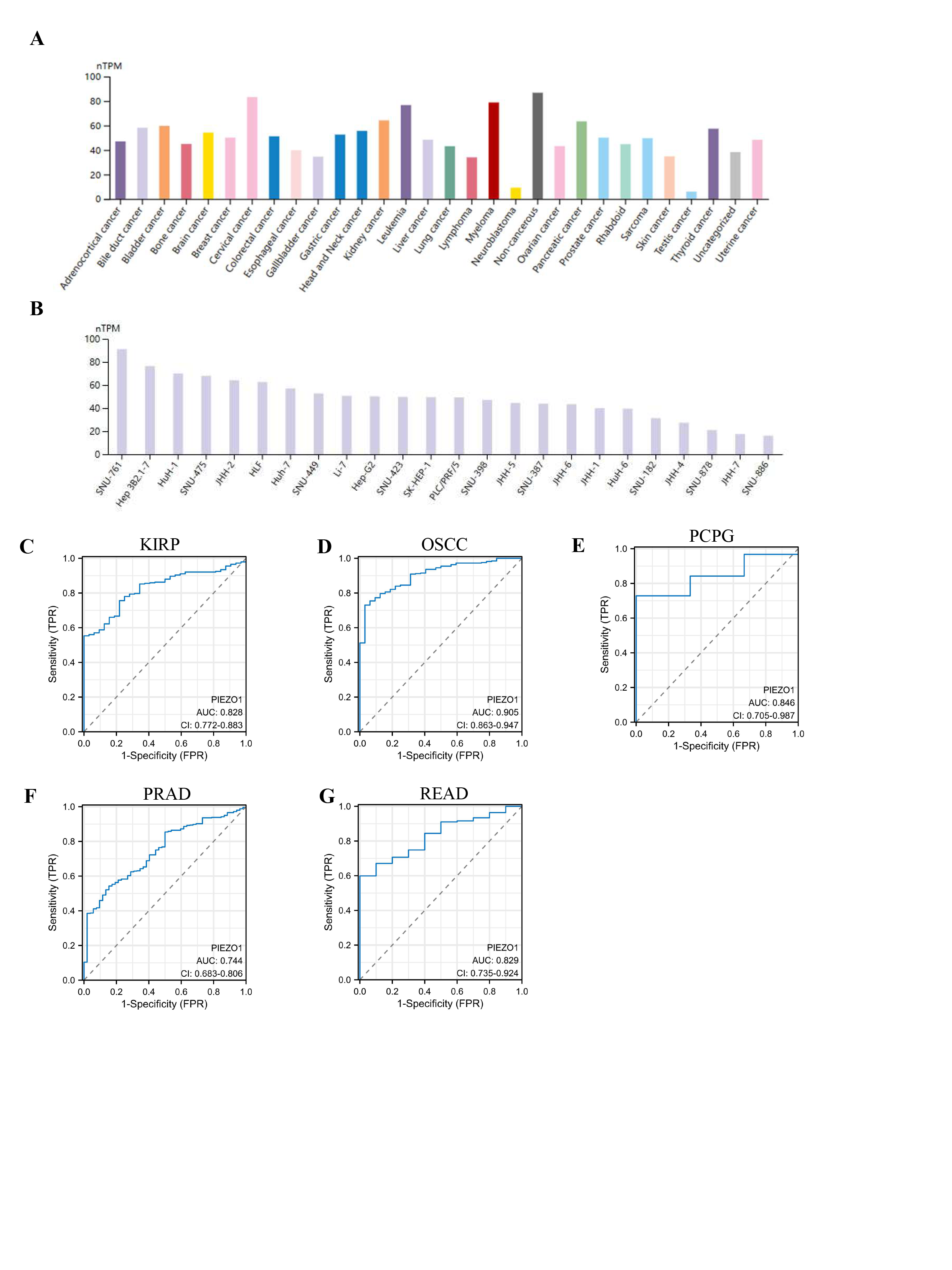

Supplement: Supplementary Figure 1 — PIEZO1 expression patterns and diagnostic performance across cancer types. (A) PIEZO1 mRNA expression across various cancer cell lines based on the HPA database. (B) PIEZO1 mRNA expression across various liver cancer cell lines based on the HPA database. (C–G) The diagnostic value of PIEZO1 was determined using ROC curves. [file Image1.tif]

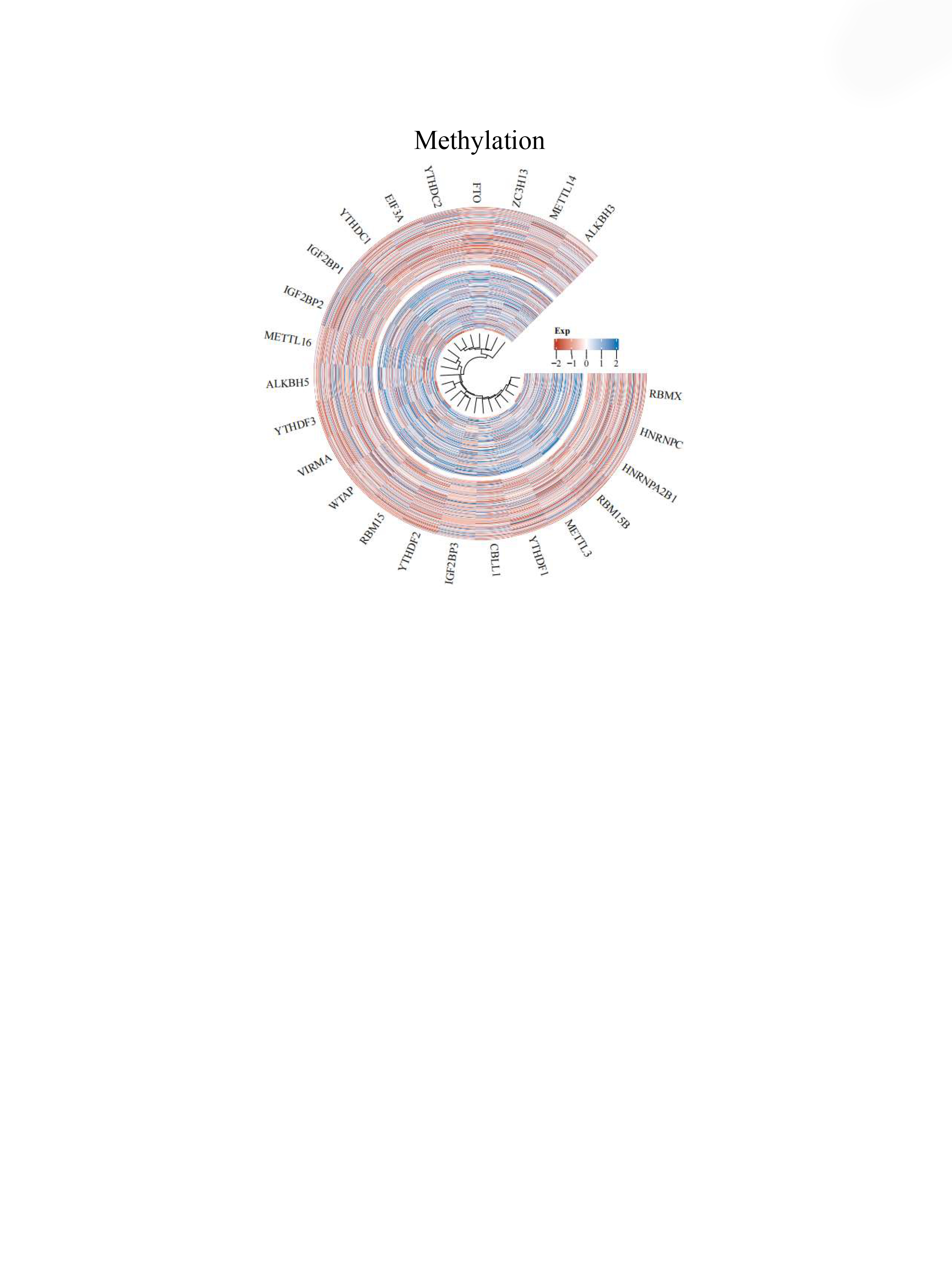

Supplement: Supplementary Figure 6 — Relationship between PIEZO1 and m6A-related genes. The correlation between PIEZO1 expression and the majority of m6A-related genes in LIHC. [file Image6.jpeg]

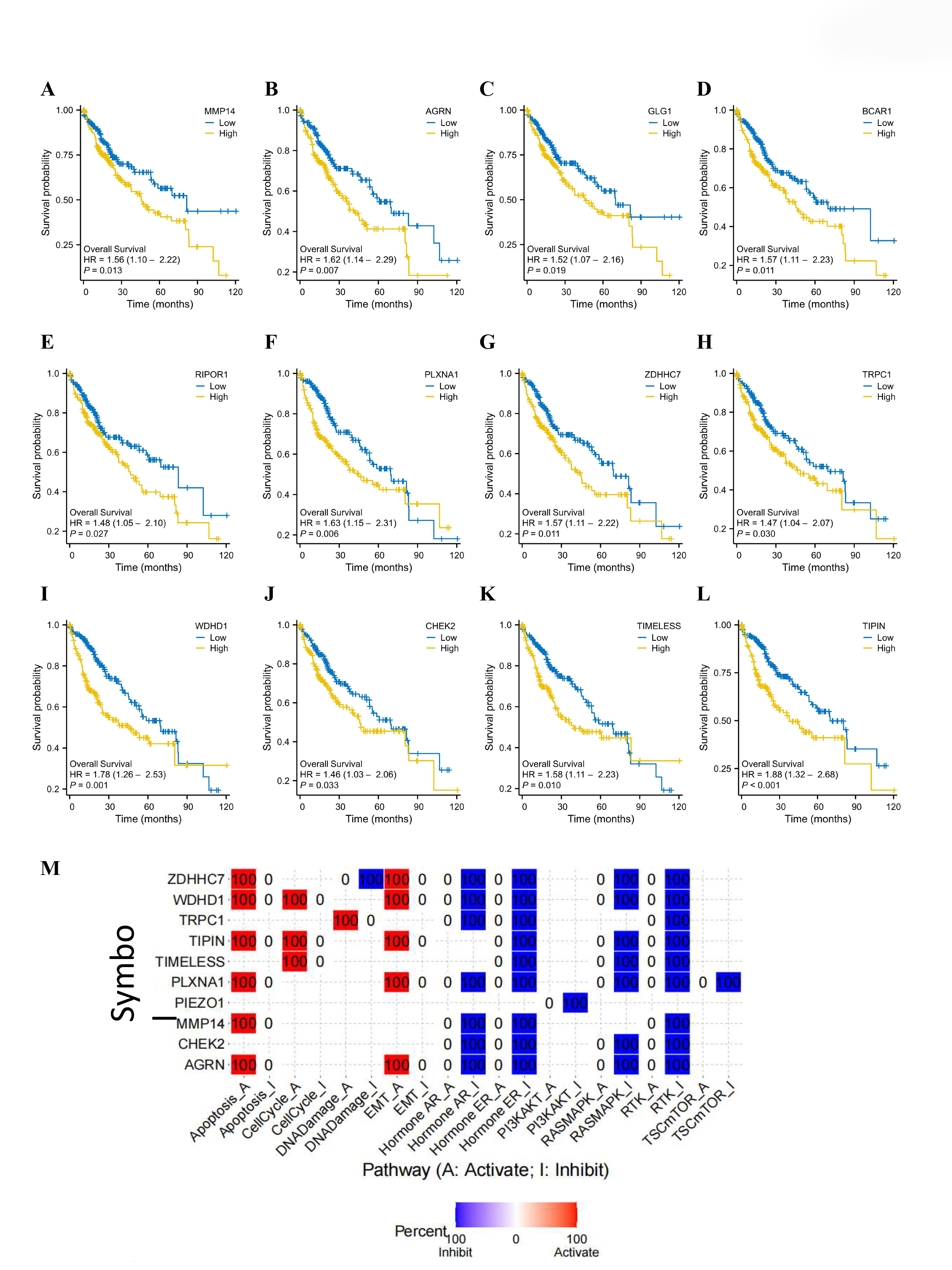

Supplement: Supplementary Figure 7 — The survival and pathway analysis of co-expression partners of PIEZO1. (A–L) Kaplan-Meier analysis was used to detect the correlations between co-expression partners of PIEZO1 and OS in LIHC. (M) The pathway analysis of PIEZO1 and its co-expressed partners in LIHC using the GSCALite tool [file Image7.jpeg]

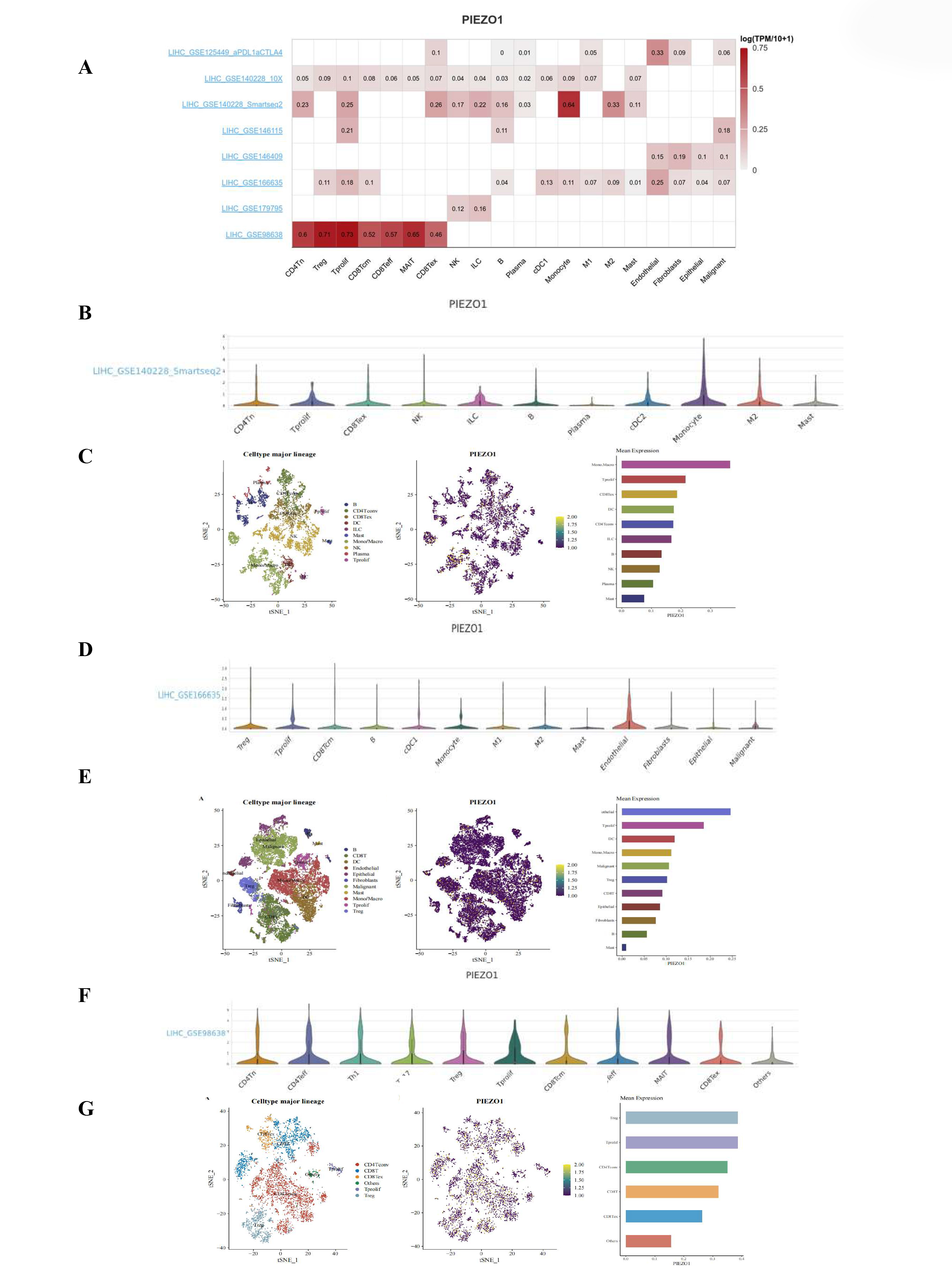

Supplement: Supplementary Figure 8 — Distribution and expression of PIEZO1 at the single cell. (A) Distribution and expression of PIEZO1 at the single cell in different LIHC databases using the TISCH2 tool. (B–G) Distribution and expression of PIEZO1 at the single cell in LIHC GSE140228 Smartseq2 (B, C), LIHC GSE166635 (D, E), and LIHC GSE98638 (F, G). [file Image8.jpeg]
